# Gluconeogenesis and PEPCK are critical components of healthy aging and dietary restriction life extension

**DOI:** 10.1371/journal.pgen.1008982

**Published:** 2020-08-25

**Authors:** Brian Onken, Natallia Kalinava, Monica Driscoll

**Affiliations:** Department of Molecular Biology and Biochemistry Rutgers University, Piscataway, NJ, United States of America; University of California San Francisco, UNITED STATES

## Abstract

High glucose diets are unhealthy, although the mechanisms by which elevated glucose is harmful to whole animal physiology are not well understood. In *Caenorhabditis elegans*, high glucose shortens lifespan, while chemically inflicted glucose restriction promotes longevity. We investigated the impact of glucose metabolism on aging quality (maintained locomotory capacity and median lifespan) and found that, in addition to shortening lifespan, excess glucose negatively impacts locomotory healthspan. Conversely, disrupting glucose utilization by knockdown of glycolysis-specific genes results in large mid-age physical improvements via a mechanism that requires the FOXO transcription factor DAF-16. Adult locomotory capacity is extended by glycolysis disruption, but maximum lifespan is not, indicating that limiting glycolysis can increase the proportion of life spent in mobility health. We also considered the largely ignored role of glucose biosynthesis (gluconeogenesis) in adult health. Directed perturbations of gluconeogenic genes that specify single direction enzymatic reactions for glucose synthesis decrease locomotory healthspan, suggesting that gluconeogenesis is needed for healthy aging. Consistent with this idea, overexpression of the central gluconeogenic gene *pck-2* (encoding PEPCK) increases health measures via a mechanism that requires DAF-16 to promote *pck-2* expression in specific intestinal cells. Dietary restriction also features DAF-16-dependent *pck-2* expression in the intestine, and the healthspan benefits conferred by dietary restriction require *pck-2*. Together, our results describe a new paradigm in which nutritional signals engage gluconeogenesis to influence aging quality via DAF-16. These data underscore the idea that promotion of gluconeogenesis might be an unappreciated goal for healthy aging and could constitute a novel target for pharmacological interventions that counter high glucose consequences, including diabetes.

## Author summary

It is known that high levels of dietary sugar can negatively impact human health, but the mechanisms underlying this remain unclear. Here we use the facile *Caenorhabditis elegans* genetic model to extend understanding of the impact of glucose and glucose metabolism on health and aging. We show that the two opposing glucose metabolism pathways–glycolysis and gluconeogenesis–have dramatically opposite effects on health: glycolytic activity responsible for sugar catabolism is detrimental, but driving gluconeogenesis promotes healthy aging. The powerful longevity regulator DAF-16 is required for the healthspan effects of gluconeogenesis. Our data highlight the intriguing possibility that driving the biosynthetic gluconeogenesis pathway could be a novel strategy for healthspan promotion. Indeed, we find that increasing levels of the core gluconeogenic enzyme PEPCK (PCK-2) in just a few intestinal cells can increase overall health in a DAF-16-dependent manner. Dietary restriction, which can promote health and longevity across species, increases PCK-2 levels in the intestine via DAF-16, and PCK-2 is required for the health benefits seen when calories are limited. Our results define gluconeogenic metabolism as a key component of healthy aging, and suggest that interventions that promote gluconeogenesis may help combat the onset of age-related diseases, including diabetes.

## Introduction

How to live at peak health over adult life is a challenge of our time. Successful aging, defined by some as the prolonged maintenance of physical and cognitive function coupled with avoidance of debilitating disease, can most certainly be influenced by activity and diet [1]. At odds with the goal of healthy aging, the “American diet” has been criticized for high sugar and high fat content that promotes muscular, cardiac, immune, and neuronal decline [2–8]. Diets rich in carbohydrates, which readily break down to glucose, can exacerbate diabetes health risks [9] and lead to sarcopenic muscle mass loss [10–12]. Although epidemiological studies support that high sugar diets impair health, tightly controlled studies of metabolic influences on aging quality are difficult to conduct in the human population, which is heterogeneous in genetics, activity, and food consumption patterns.

Animal models have been highly instructive regarding conserved metabolic states that promote or defer age-associated decline. In the facile aging model *Caenorhabditis elegans*, high glucose in growth medium shortens lifespan independently of associated osmotic change or glucose metabolism by bacterial food, in part via insulin-like signaling [13–20]. The insulin/IGF-1 receptor ortholog DAF-2 activates the AGE-1 PI3 kinase to signal downstream to inhibit the activity of longevity-promoting transcription factors including DAF-16/FOXO. Glucose stimulates insulin signaling to decrease lifespan in part by inhibiting DAF-16 [16, 18, 19]. Conversely, low insulin signaling activity activates DAF-16-mediated transcription to promote longevity [21–28].

Restricting glucose use can confer health benefits. For example, limiting *C*. *elegans* glucose catabolism by administering non-metabolizable 2-deoxyglucose results in longevity [19, 29]. More generally, dietary restriction (DR), a physiological state induced by reduced caloric intake, increases lifespan across species. In mammals, DR reduces blood glucose and insulin levels, and decreases glycolytic gene expression [30–32]. In *C*. *elegans*, complex pathways can induce DR benefits of longevity and prolonged vigor [33–43]. Still, several methods of food limitation extend lifespan via a DAF-16-dependent mechanism, suggesting that DR increases longevity by engaging a mechanism linked to the insulin signaling pathway under at least some conditions [34, 38, 40, 43].

How glucose impacts tissue-specific features of age-associated decline, such as sarcopenic loss of muscle mass and strength, is a central issue in healthy maintenance that remains poorly understood. Likewise, metabolic circuits linking glucose, DR, and insulin signaling remain to be fully elaborated [17]. To begin to address these relationships, we examined the impact of genetically perturbing glycolytic and gluconeogenic flux on maintained *C*. *elegans* muscle function and on survival during adult life.

Here we report on how glucose metabolism intersects with longevity pathways to influence aging quality. We show that high glucose levels and high glycolytic activity negatively impact adult locomotory and general health, while conversely, gluconeogenic activity promotes maintenance of healthy muscle function and mid-life survival. Conserved transcription factor DAF-16 is crucial for these effects: glycolysis inhibits DAF-16 to compromise healthspan, while DAF-16 signaling directly promotes gluconeogenic gene expression to extend healthspan. DAF-16-dependent expression of gluconeogenesis-specific enzyme PEPCK PCK-2 in specific intestinal cells is critical for this regulation, suggesting that metabolic regulation in specific cells drives animal-wide health. Multiple DR pathways elevate gluconeogenic gene expression via DAF-16, and we show this gluconeogenic gene expression is required for extended healthspan under DR. Our results demonstrate that interventions that promote gluconeogenic metabolism can improve overall health, possibly by inducing a DR-like physiological state. We suggest that interventions that promote gluconeogenesis constitute a novel strategy for combating age-associated diseases and accelerated aging-related glucose homeostasis dysregulation.

## Results

### High glucose accelerates sarcopenic decline of muscle-controlled *C*. *elegans* behaviors

Elevated glucose systemically impairs human health with consequences particularly potent in middle to older age. The mechanisms by which high glucose compromises individual tissue functions remain poorly understood at the molecular level. In particular, how high glucose diets relate to conserved degenerative processes such as sarcopenia—the progressive loss of muscle mass and muscle strength over time that lowers quality of life and increases frailty—is unclear.

*C*. *elegans* locomotion rates decline with age in a manner that is roughly correlated with the degree of sarcopenic muscle deterioration [44, 45] but that also involves neuronal health [46, 47]. Cellular phenotypes associated with human muscle skeletal decline, including disorganization and loss of sarcomeres as well as fat infiltration of muscle, are observed in bodywall muscles of aging nematodes that progressively slow in locomotory capacity. Likewise, *C*. *elegans* cardiac-like muscle that makes up the pharynx undergoes significant structural and functional decline with age [45, 48, 49].

**Fig 1:**
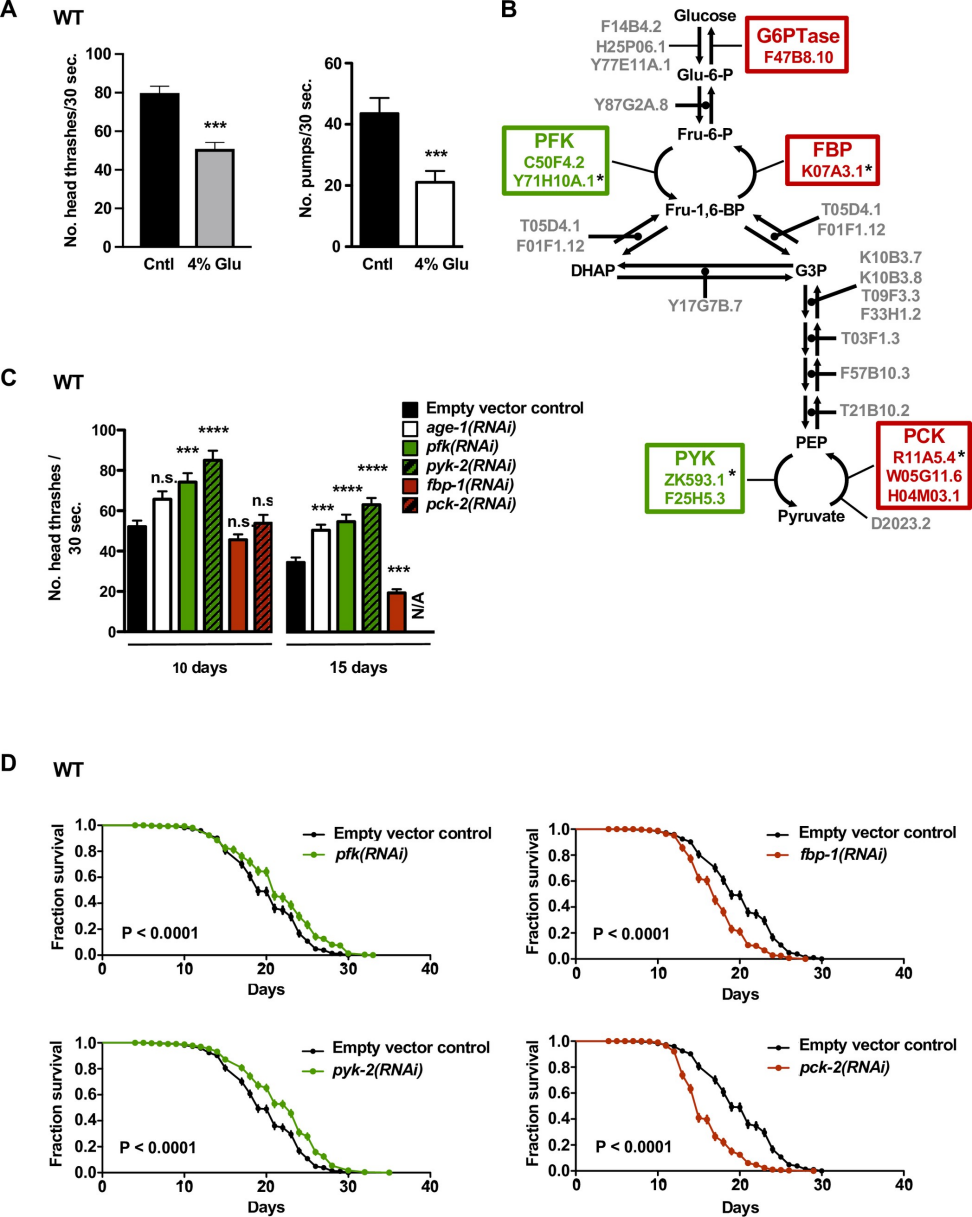
Disrupting Glycolysis-promoting Genes Extends *C*. *elegans* Healthspan, Whereas High Glucose and Gluconeogenic Gene Disruptions Shorten Healthspan. (A) Left-hand graph: swimming rates for WT animals raised on control media or media containing 4% glucose. We recorded the number of head bends (i.e. head “thrashes”) / 30 seconds for individual animals placed in liquid media on day 8 of life. 4% glucose significantly decreases WT locomotory ability (*** *P* = 0.0005, unpaired t test; averages from three trials, n = 30 animals per condition; error bars represent SEM). Trials with 5 and 13 day old animals also showed impaired older age locomotion in 4% glucose (Supplemental S1A and S1B Fig). Right-hand graph: We recorded pharyngeal pumping of individual wild-type 5 day old animals raised from the egg stage on control media or media containing 4% glucose; 30 second records. 4% glucose significantly impairs pumping rate (*** P = 0.0008, unpaired t test, n = 35 animals per condition, error bars represent SEM; averages from three trials per condition). (B) *C*. *elegans* glycolytic and gluconeogenic pathways. Although most enzymes of glycolysis catalyze both forward (green, glycolysis) and reverse (red, gluconeogenesis) reactions, PFK/FBP and PYK/PCK are enzyme pairs that catalyze one-way, opposing reactions: glycolytic enzymes PFK (phosphofructokinase) and PYK (pyruvate kinase) and gluconeogenic enzymes FBP (fructose-1,6-bisphosphatase) and PCK (phosphoenolpyruvate carboxykinase). Glu-6-P = glucose-6-phosphate; Fru-6-P = fructose-6-phosphate; Fru-1,6-BP = fructose-1,6-bisphosphate; DHAP = dihydroxyacetone phosphate; G3P = glyceraldehyde-3-phosphate; PEP = phosphoenolpyruvate. * Gene disruptions studied most extensively in this work (*C*. *elegans* ORF designations indicated). We note that, in our hands, disruption of C50F4.2 (*pfk-1*.*2*), F25H5.3 (*pyk-1*), and W05G11.6 (*pck-1*) did not impact lifespan. Throughout the paper, data on genes that normally promote glycolysis are represented in green; data on genes that normally promote gluconeogenesis are presented in red. (C) Swimming rate profiles for WT animals treated with glycolysis-limiting *pfk(RNAi)* and *pyk-2(RNAi)* or gluconeogenesis-limiting *fbp-1(RNAi)* and *pck-2(RNAi)*. *age-1(RNAi)* is used as a positive control known to extend old age locomotory ability. Animals treated with glycolysis-limiting *pfk(RNAi)* and *pyk-2(RNAi)* swim significantly better than controls on days 10 and 15 of life; (*pfk(RNAi)* confers a 42% increase in swimming ability on day 10 and a 59% increase on day 15; *pyk-2(RNAi)* confers a 63% increase on day 10 and an 83% increase on day 15). It may be worth noting that these increases we recorded in this experiment are higher than those seen for the *age-1* positive control RNAi (for *age-1(RNAi)*, a 26% and a 46% increase in swimming ability on days 10 and 15, respectively, in this study) so locomotory impact appears relatively substantial, assuming *age-1(RNAi)* efficacy. On day 15, *fbp-1(RNAi)* decreases swimming rates by 44%, and *pck-2(RNAi)* animals moved too slowly to measure (or were dead) (marked as N/A). Data shown are pooled from two similar independent trials, n = 40 animals per condition per trial. Error bars represent SEM. *** *P* < 0.001; **** *P* < 0.0001; n.s. = not significant; all statistical analyses by one way ANOVA with Dunnett’s multiple comparisons test; N/A = not available. (D) Survival curves of WT animals treated with *pfk(RNAi)*, *pyk-2(RNAi)*, *fbp-1(RNAi)*, and *pck-2(RNAi)*. Animals treated with RNAi targeting glycolytic genes *pfk* and *pyk-2* show increases in median survival (an 8.85% increase for *pfk* disruption and a 20.83% increase for *pyk-2* disruption) and have significantly increased survival relative to controls (*P* < 0.0001 for both *pfk* and *pyk-2* disruptions, Log-rank test), although maximal lifespan is not significantly affected (Supplemental S1D Fig). Conversely, animals treated with RNAi directed against gluconeogenic genes *fbp-1* and *pck-2* have lowered median survival (a 10.94% decrease for *fbp-1* disruptions and a 21.88% decrease for *pck-2* disruptions) and exhibit lower survival relative to controls (*P* < 0.0001 for both *fbp-1* and *pck-2* disruptions, Log-rank). Animals treated with the *age-1(RNAi)* positive control show a 46.88% increase in median survival and significantly increased survival (*P* < 0.0001, Log-rank; refer to S1 Table for survival data with *age-1(RNAi)*). Data shown are pooled from 6 independent trials, n = 60 animals per condition per trial.

To examine the impact of high glucose on locomotory ability, we exposed wild-type *C*. *elegans* to increasing levels of glucose and recorded swim vigor in older age. (Swim behavior is directed by worm skeletal muscle-like bodywall muscle [50, 51]; here and throughout the text we use a swim vigor measure (number of head bends/30 sec.) as a measure of locomotion capacity). We found that 4% glucose media impairs swimming ability of young adults (5 days old, Supplemental S1A Fig); middle-aged adults (Fig 1A, left-hand graph, 8 days old), and animals of more advanced age (13 days old, Supplmental Fig 1B); 2% glucose, which has been shown to decrease lifespan [16, 20, 52], did not impact swimming in our hands (Supplemental S1C Fig). In addition, we found that exposure to high glucose reduces older age pharyngeal pumping mediated by cardiac-like pharyngeal muscle (Fig 1A, right-hand graph; 5 day old measure scored because pharyngeal muscle function declines faster than bodywall muscle). Together, these outcomes reveal a progeric influence of high glucose on physical activity and muscle function reminiscent of that suggested for glucose impact on muscle-associated decline in human elderly [11, 12, 53] and consistent with recent studies of glucose toxicity on old-age mobility [13, 20].

### Glucose catabolism is associated with accelerated aging traits

Aging induces glycolytic gene expression in mammals [30, 32], and, in *C*. *elegans* under standard feeding conditions, inhibiting glucose catabolism with the glucose analog 2-deoxyglucose or with genetic inhibition of at least one glycolytic enzyme (pyruvate kinase, or PYK) promotes longevity [19, 52]. In addition, treatment with the glycolysis intermediate DHAP has a large detrimental impact on *C*. *elegans* lifespan [52]. These observations suggest that the process, or consequences, of glucose utilization could be linked to age-associated decline in locomotion.

To address how glucose catabolism influences functional aging under standard feeding conditions, we disrupted the specific *C*. *elegans* enzymes that catalyze unidirectional, irreversible steps in glycolysis. Although most reactions of glycolysis are readily reversible, distinct enzymes execute the forward and reverse reactions for the fructose-6-phosphate/fructose 1,6 bisphosphate interconversion (PFK/FBP) and the P-enolphosphate/pyruvate interconversion (PYK/PCK) (see Fig 1B). We anticipated that RNAi-mediated disruption of the reactions that specifically promote glycolysis (*i*.*e*., enzymes PFK and PYK) should have a predominant metabolic effect of limiting glycolytic flux, as appears to be the case for 2-deoxyglucose [19, 29]. Indeed, we found that knockdown of the glycolysis-promoting phosphofructokinase (*pfk1*.*1*, hereafter referred to as *pfk*) and pyruvate kinase (*pyk-2*) genes results in significant increases in swim ability in mid- and late life (Fig 1C). We conclude that limiting glycolysis can robustly protect against age-associated mobility decline, consistent with a previous report [20]. Furthermore, we find that disruptions of glycolytic genes *pfk* and *pyk-2* also result in increases in median survival (Fig 1D and S1 Table), supporting that limiting glycolysis promotes overall health in aging adult populations.

It is interesting to note that the gains in swimming ability and median survival we observe with unidirectional glycolysis gene disruptions are not accompanied by comparably large increases in maximal lifespan (Supplemental S1D Fig). This observation is important in light of the study of Bansal et al. [54] that suggests that some long-lived *C*. *elegans* mutants spend extended periods of time in a frail state at the end of life, in proportion to their increased maximal lifespan. The glycolysis interventions we highlight appear to maintain youthful physiology in mid-life without extending late-life frailty. The inability of glycolysis interventions to extend maximal lifespan may indicate midlife-specific metabolic consequences, or alternatively might be attributed to confounding failures in older animals not improved by glycolytic inhibition. Blocking glycolysis may result in the build-up of glucose, which could reach toxic levels later in life. In sum, knockdown of enzymes that specifically catalyze one-way steps critical for glucose breakdown can improve multiple measures of healthy aging, supporting that glycolytic flux confers an overall negative impact on physiology that promotes functional aging.

### Inhibiting unidirectional glycolysis-promoting reactions increases mid-life health via DAF-16

**Fig 2:**
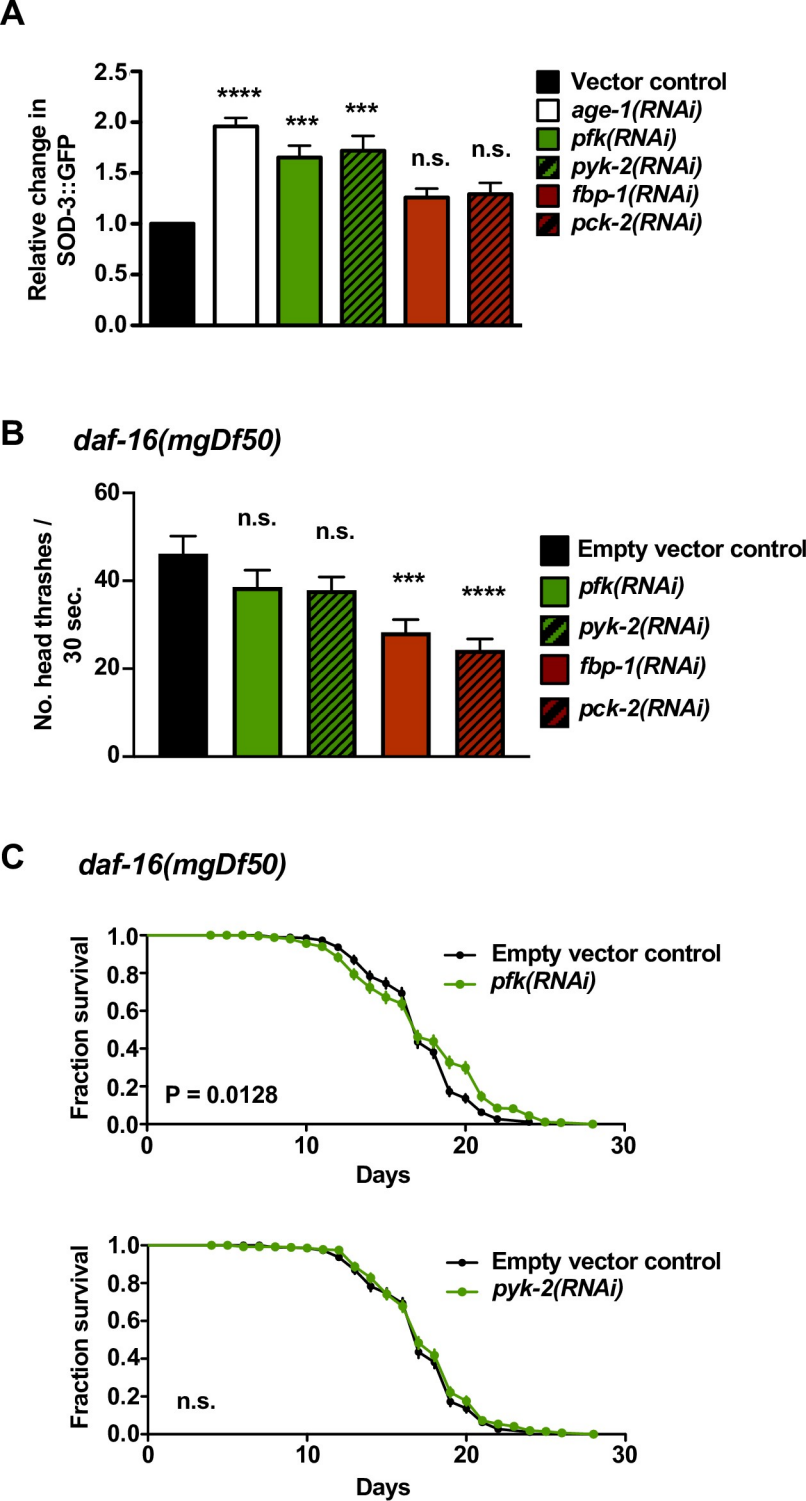
DAF-16/FOXO is Required for Locomotory Healthspan and Lifespan Benefits of Genetic Glycolysis Disruption. (A) Fluorescence intensity levels of wild-type animals expressing a GFP transcriptional reporter for *sod-3*, which expresses superoxide dismutase and is a direct transcriptional target of DAF-16. We measured *in vivo* fluorescence using a spectrofluorimeter on day 5 of life. As expected from previous work [74], decreased insulin signaling with *age-1(RNAi)* results in increased SOD-3::GFP expression. Similarly, knockdown of glycolytic genes via *pfk(RNAi)* and *pyk-2(RNAi)* significantly increases SOD-3::GFP fluorescence levels. Disrupting the *fbp-1* and *pck-2* gluconeogenic genes, on the other hand, does not significantly change GFP fluorescence levels. Data are pooled averages from 5 independent trials, n = 50 animals per condition per trial. Error bars represent SEM. *** *P* < 0.001; **** *P* < 0.0001; n.s. = not significant; all by one way ANOVA with Dunnett’s multiple comparisons test. (B) Swimming rates of *daf-16(mgDf50)* mutants treated with glycolytic and gluconeogenic RNAi. Disrupting glycolysis-promoting genes *pfk* or *pyk-2* in the *daf-16* null background eliminates old-age benefits that occur with *pfk(RNAi)* or *pyk-2(RNAi)* (refer to Fig 1C), indicating a requirement for *daf-16* in the benefits of glycolysis impairment. In contrast, disruption of gluconeogenic genes *fbp-1* or *pck-2* results in significant decreases in swimming rates in *daf-16* animals, similar to wild-type. Data shown are pooled from two independent trials, n = 40 animals per condition per trial. Measurements were taken on day 10 of life. Error bars represent SEM. *** *P* < 0.001; **** *P* < 0.0001; n.s. = not significant; all by one way ANOVA with Dunnett’s multiple comparisons test. *daf-16* mutants are documented to have lowered locomotory ability vs. WT later in life [91, 92]; we note that, although comparison of scores across experiments is not possible due to variation in baseline values, the swimming rate of *daf-16(mgDf50)* control animals was slightly lower than WT controls on the same day of life (in Fig 1C; mean of 46.17 head thrashes/30 sec. for *daf-16* vs. 52.17 head thrashes/30 sec. for WT on day 10). (C) Survival curves of *daf-16(mgDf50)* null mutants treated with RNAi against glycolytic genes *pfk* and *pyk-2*. The beneficial effects of glycolytic gene disruptions seen in wild-type animals (refer to Fig 1D) are mostly eliminated in the *daf-16* mutant background: median survival is not increased in *daf-16* animals treated with either RNAi, and the survival curve of *daf-16* mutants raised on *pyk-2(RNAi)* is not significantly (n.s.) different than controls (Log-rank test). *pfk(RNAi)* slightly increases the survival of *daf-16* mutants later in life (as indicated by a right-shifted survival curve in late-life; Log rank calculated *P* = 0.0128), suggesting possible *daf-16*-independent mechanisms for lifespan extension in older animals with glycolytic inhibitions. Data are pooled from 5 independent trials, n = 60 animals per condition per trial, details in S1 Table.

DAF-16/FOXO is a critical downstream transcription factor in longevity-promoting pathways [22], and inhibition of DAF-16 activity has been previously implicated in the glucose toxicity mechanism [16, 19]. We therefore tested whether DAF-16/FOXO might act to promote healthy aging when glycolysis is genetically impaired. We first sought evidence of DAF-16 activation under conditions of *pfk* and *pyk-2* RNAi using a well-characterized reporter of DAF-16 activity, SOD-3::GFP, a direct transcriptional target of *C*. *elegans* DAF-16 [55, 56]. We found that both *pfk(RNAi)* and *pyk-2(RNAi)* result in significant increases in SOD-3::GFP levels (Fig 2A), suggesting that glycolytic gene disruptions may generally increase DAF-16 transcriptional activity. Indeed, *daf-16* is critical for the strong healthspan effects of glycolytic gene disruptions we document in Fig 1: the large increases in swimming ability and the median survival extension of wild-type animals treated with RNAi against glycolytic genes *pyk-2* and *pfk* are largely eliminated in *daf-16* null mutants (Fig 2B and 2C and S1 Table, overall survival analyses; we note that, although median survival does not increase with *pfk(RNAi)* in the *daf-16* mutant background, the *daf-16* lifespan curve is significantly right-shifted late in life with *pfk(RNAi)* treatment, suggesting that glycolytic interventions may impact longevity independently of DAF-16 in older animals).

If DAF-16 were activated by knockdown of glycolytic *pfk* and *pyk-2* via an insulin signaling pathway, we might expect that *pfk* and *pyk-2* disruption in an insulin pathway activation background would not further enhance health and lifespan phenotypes. We tested this possibility by performing *pfk* and *pyk-2* RNAi in the *age-1* insulin signaling pathway mutant, well characterized for activated DAF-16 and associated longevity [26]. We found that disrupting glycolytic *pfk* or *pyk-2*, which extend locomotory healthspan and mid-life survival in wild type (Fig 1C and 1D and S1 Table), does not enhance locomotory ability in the *age-1* mutant background (S2A Fig) and *pfk(RNAi)* does not significantly improve *age-1* lifespan curves (S1 Table). The lack of benefits of *pfk* and *pyk-2* knockdown in *age-1* is consistent with a model in which *pfk* and *pyk-2* disruption can activate DAF-16, possibly via a common insulin signaling pathway: enhanced DAF-16 activity can bypass deleterious consequences of glycolysis. We note that *pyk-2(RNAi)* can extend *age-1* lifespan (S1 Table), suggesting that the glycolytic pathway may also influence lifespan via pathways other than the insulin pathway and DAF-16 (as supported by our results with *pfk(RNAi)* in the *daf-16* background, described above).

Together these data on *daf-16* demonstrate that inhibiting unidirectional glycolytic enzymes increases locomotory healthspan and median lifespan via a DAF-16-dependent mechanism. Data also support that modulation of insulin signaling is associated with glycolytic flux changes, consistent with previous studies on *C*. *elegans* glucose toxicity [13, 16–20].

### Reversing glycolytic flux (*i*.*e*., promoting gluconeogenesis) promotes healthy aging

The existence of enzymes that specifically catalyze the reverse reactions of PFK and PYK-2 enabled us to test whether disruption of gluconeogenesis promotes effects opposite to the positive outcomes of RNAi-mediated glycolysis inhibition. We found that animals treated with RNAi against genes that encode the unidirectional gluconeogenic enzymes fructose-1,6-bisphosphatase (FBP-1), which catalyzes the gluconeogenic reaction reverse to PFK, and phosphoenolpyruvate carboxykinase (PCK-2), which catalyzes the gluconeogenic reaction reverse to PYK-2 (Fig 1B), exhibit major impairments in locomotory ability in late life (disrupting *pck-2* knockdown has such strong effects that animals were moving too slowly (or too few animals remained alive) to measure on day 15) (Fig 1C). Disrupting gluconeogenic genes *fbp-1* and *pck-2* also significantly shortens median lifespan (Fig 1D and S1 Table).

To rule out that *pck-2(RNAi*) might merely confer general sickness, we tested *pck-2* inhibition in the *age-1* background. We find that *pck-2(RNAi*) does not reduce locomotory rates or lifespan extension in the *age-1* background (S2A and S2B Fig and S1 Table), supporting that impairing gluconeogenic activity does not universally compromise health. We conclude that *fbp-1* and *pck-2* dependent reactions that promote gluconeogenesis are needed to maintain normal adult healthspan. Our findings indicate that gluconeogenesis pathways are critical for late life health under standard growth conditions, and constitute the first focused demonstration that *fbp-1* and *pck-2* deficiency can be progeric for *C*. *elegans*.

In sum, inhibiting the enzymes that specifically promote glycolysis confers beneficial effects on both locomotory healthspan and median survival, whereas inhibiting enzymes that specifically promote gluconeogenesis negatively impacts these two indicators of adult health. Data suggest that glycolysis is deleterious, and gluconeogenesis is beneficial for healthy aging, especially as judged by locomotory capacity.

### Overexpression of gluconeogenic PEPCK-C gene *pck-2* extends locomotory healthspan and lifespan

Phosphoenolpyruvate carboxykinase (PEPCK) catalyzes the rate-controlling step of gluconeogenesis, and is thus a central player in glucose homeostasis. In mammals, there are two forms of the enzyme: cytosolic PEPCK-C and mitochondrial PEPCK-M, with PEPCK-C thought to play the larger role in metabolic regulation [57]. Given the striking requirement for gluconeogenic *pck-2* in maintained locomotory health and normal lifespan (Fig 1C and 1D and S1 Table) and previous indications that PEPCK can drive metabolic states [58, 59], we focused on mechanisms of *pck-2* contributions to adult health (*pck-2* does not include a detectable mitochondrial targeting sequence and PCK-2 expressed from a rescuing transgene does not localize to mitochondria (S3A Fig), supporting *pck-2* encodes a PEPCK-C; in our hands, sequence-confirmed RNAi knockdown of *pck-1*, which encodes another *C*. *elegans* PEPCK ortholog [59] did not impact aging, S1 Table).

Since disruption of *pck-2* is deleterious to health, we first asked whether elevated *C*. *elegans* PEPCK expression might suffice to promote healthy metabolism associated with enhanced late adult maintenance. We examined a strain carrying multiple integrated copies of *P*_*pck-2*_*pck-2*::*gfp* for potential benefits of *pck-2* over-expression in healthy aging. We found that the Is[*P*_*pck-2*_*pck-2*::*gfp*] over-expression line exhibits increases in midlife locomotory ability and median survival (but not maximal lifespan) as compared to controls that express only GFP from the *pck-2* promoter (S3B and S3C Fig and S1 Table; the promoter-only Is[*P*_*pck-2*_*gfp*] control construct had no effect on wild-type locomotory ability or lifespan, S3C, S3D and S3E Fig and S1 Table). We conclude that over-expression of *pck-2*, which encodes the unidirectional, rate-limiting enzyme of gluconeogenesis, can extend locomotory healthspan and median lifespan.

### *pck-2* overexpression lifespan extension requires gluconeogenesis

**Fig 3:**
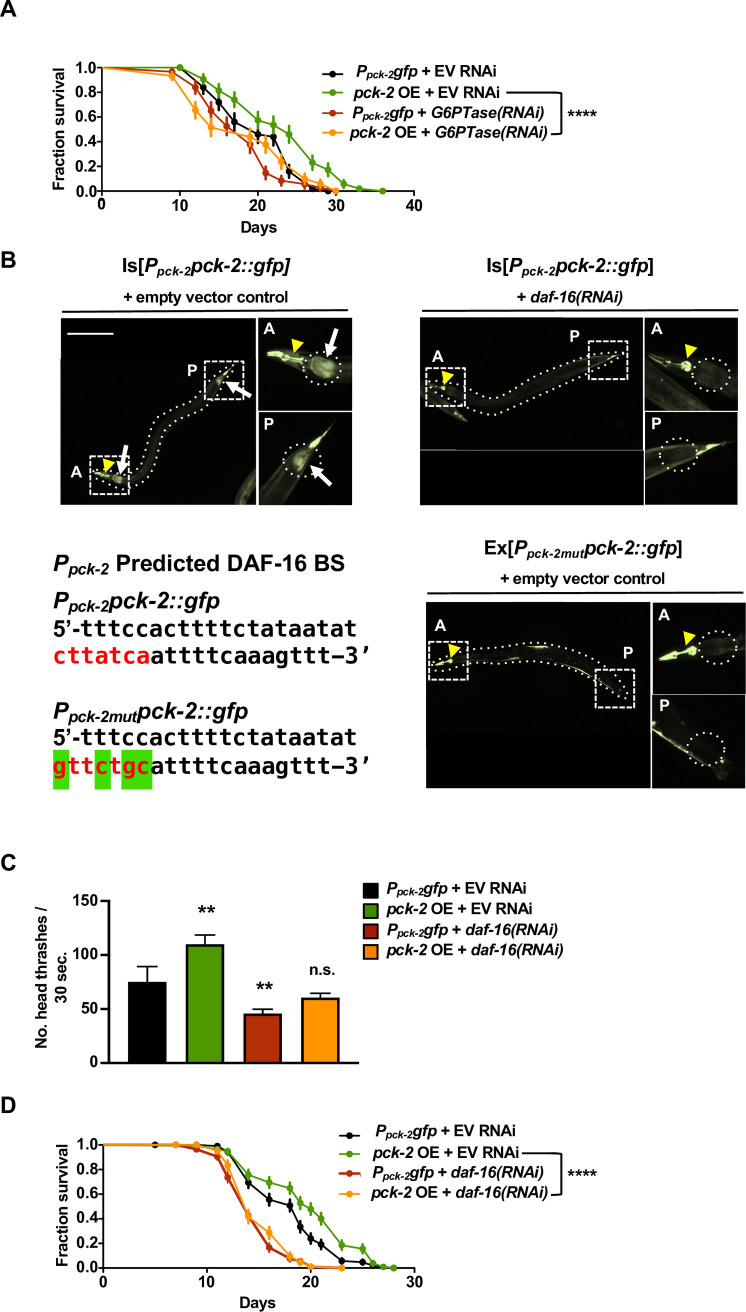
Increased Locomotory Healthspan and Survival with *pck-2* Overexpression, as Well as Expression of *pck-2* in the Intestine, Require *daf-16*. (A) Survival curves of wild-type animals expressing a transcriptional reporter for *pck-2* (*P*_*pck-2*_*gfp*, used as the control strain) or the intact *pck-2* expressed from its own promoter (*P*_*pck-2*_*pck-2*::*gfp*, “*pck-2* OE”, used as the *pck-2* over-expressor strain) and treated with empty vector control (EV RNAi) or *F47B8*.*10(RNAi)*. *F47B8*.*10* encodes glucose-6-phosphate translocase (G6PTase), an enzyme that functions in a complex with G6P phosphatase to catalyze the final step of gluconeogenesis in vertebrates [61]. *pck-2* overexpression significantly increases survival (*P* = 0.0020, Log-rank) that depends on G6PTase (survival curves of control animals and *pck-2* over-expressors treated with *F47B8*.*10/G6PTase(RNAi)* are not significantly different (n.s., Log-rank)). Data shown are from three trials, n = 60 animals per condition. (B) Wild-type animals expressing *pck-2*::*gfp* from the native *pck-2* promoter (Is[*P*_*pck-2*_*pck-2*::*gfp*]) and treated with an empty vector control RNAi display a GFP signal in cells in the anterior (“A”) and posterior (“P”) intestine (arrows, top left panels; day 7 of life; approximately 35 out of 60 animals displayed this intestinal GFP pattern on day 7) as well as in the pharynx (arrowhead; all animals displayed pharyngeal GFP on day 7). *daf-16(RNAi)* treatment specifically eliminates the intestinal GFP signal (top right-hand panels; no intestinal GFP was detected in any of the 60 observed animals on day 7). The predicted DAF-16 binding site in the *pck-2* promoter is shown on the bottom left in red; green highlighting indicates altered base pairs in the binding site mutant. Disruption of the DAF-16 binding site 1086 base pairs upstream of the *pck-2* start codon [63] in the *pck-2* promoter in Ex[*P*_*pck-2mut*_*pck-2*::*gfp*] (bottom panels) also eliminates the intestinal *pck-2*::*gfp* signal (bottom right; no intestinal GFP was detected in any of the 60 observed animals on day 7). Bar, 200 μm. (C) Swimming rates of wild-type animals expressing either a *pck-2* transcriptional reporter that lacks any *pck-2* coding sequences (*P*_*pck-2*_*gfp*, used as the control strain) or the intact *pck-2* gene expressed from its own promoter (*P*_*pck-2*_*pck-2*::*gfp*, here indicated as “*pck-2* OE”, used as the *pck-2* over-expressor strain) treated with an empty vector control RNAi (EV RNAi) or *daf-16(RNAi)*. *pck-2* overexpression (OE) significantly increases swimming rates vs. wild-type controls (*P* < 0.01, one way ANOVA). Swimming fitness is disrupted with *daf-16(RNAi)* in both WT controls and *pck-2* OE animals (*P* < 0.01, one way ANOVA). Swimming rates of control and *pck-2* OE animals treated with *daf-16(RNAi)* are not significantly different from one another, one way ANOVA. Data shown are from averages from three trials, n = 40 animals per condition. Measurements were taken on day 9 of life. Error bars represent SEM. ** *P* < 0.01. All one way ANOVA analyses were performed with Dunnett’s multiple comparisons test. (D) Survival curves of wild-type animals expressing a transcriptional reporter for *pck-2* (*P*_*pck-2*_*gfp*, used as the control strain) or the intact *pck-2* expressed from its own promoter (*P*_*pck-2*_*pck-2*::*gfp*, “*pck-2* OE”, used as the *pck-2* over-expressor strain) and treated with empty vector control (EV RNAi) or *daf-16(RNAi)*. *pck-2* overexpression (OE) significantly increases survival (*P* = 0.0017, Log-rank test). The *pck-2* OE survival benefit is abolished with *daf-16(RNAi)* (control and *pck-2* OE animals treated with *daf-16(RNAi)* have shortened lifespans that are not significantly (n.s.) different from one another, Log-rank test). Data shown are pooled from 2 independent trials, n = 60 animals per condition per trial. **** *P* < 0.0001. Details in S1 Table.

In mammals, PEPCK is widely expressed, and, in addition to its role in gluconeogenic tissues, PEPCK plays an anaplerotic role in non-gluconeogenic cells (i.e., in glyceroneogenesis; [57]). To ask whether *pck-2* overexpression acts via its gluconeogenesis role, we tested the requirement for the glucose-6-phosphatase complex in the improved mid-life survival seen in our *pck-2* overexpressing strain. In mammals, glucose-6-phosphatase is specifically expressed in gluconeogenic tissues where it catalyzes the final step of gluconeogenesis [60]. Here we targeted the ortholog of glucose-6-phosphate translocase, a component of the glucose-6-phosphatase complex in vertebrates ([61]; see Supplemental S4 Fig for homology alignment; note that the *C*. *elegans* glucose-6-phosphatase has not been experimentally identified). We found that the lifespan benefits of *pck-2* overexpression are absent when glucose-6-phosphate translocase is disrupted (Fig 3A and S1 Table). Thus *pck-2* overexpression acts via a gluconeogenesis pathway to improve adult health.

### *pck-2* is expressed in specific intestinal cells via a *daf-16*-dependent mechanism

To better understand how gluconeogenic PCK-2 improves adult health, we probed the expression pattern of *pck-2*. We used the Is[*P*_*pck-2*_ *pck-2*::*gfp*] reporter to determine the tissues/cells in which PCK-2 is likely needed for adult health impact. In younger animals under standard growth conditions, the Is[*P*_*pck-2*_ *pck-2*::*gfp*] translational fusion is expressed in the pharynx, in the intestine, in bodywall muscle, and in hypodermis. In adults, Is[*P*_*pck-2*_ *pck-2*::*gfp*] expression becomes strikingly restricted to the very most anterior and posterior intestinal cells (Fig 3B). We also note that, consistent with its assignment as a PEPCK-C ortholog, PCK-2::GFP appears localized to the cytoplasm in the cells in which it is expressed (S3A Fig).

Since we had determined that *daf-16* is important for the benefits associated with glycolysis downregulation (Fig 2), we wondered whether *daf-16* might be important for the *pck-2* over-expression healthspan effects. Indeed, the *pck-2* promoter includes a consensus DAF-16 binding site, and has been identified as a direct target of DAF-16 [62, 63]. To test whether DAF-16 is required for *pck-2* expression, we performed *daf-16(RNAi)* in the Is[*P*_*pck-2*_ *pck-2*::*gfp*] strain. We find that *daf-16(RNAi)* eliminates the GFP signal specifically in adult intestinal cells but not in other tissues in younger animals (Fig 3B), establishing that the intestinal expression of *pck-2* is DAF-16-dependent. To confirm direct targeting of *pck-2* by DAF-16, we altered the putative DAF-16 binding site in the *pck-2* promoter (Fig 3B) and examined Ex[*P*_*pck-2mut*_ *pck-2*::*gfp*] expression. We find that disruption of the DAF-16 consensus binding site eliminates intestinal expression, supporting that DAF-16 directly regulates *pck-2* expression in specific intestinal cells via consensus DAF-16 target sites (Fig 3B). Furthermore, the locomotory and median lifespan benefits associated with *pck-2* over-expression are completely abolished by *daf-16(RNAi)*: the mid-age health phenotypes of *pck-2* over-expressing animals under *daf-16* RNAi knockdown conditions are similar to that of control animals treated with *daf-16(RNAi)* (Fig 3C, D).

Although DAF-16 is required for increased lifespan with both reduced insulin signaling [21–28] and *pck-2* overexpression, *pck-2* is not required for the long lifespan of *age-1* insulin pathway mutants (S2B Fig), suggesting that gluconeogenic activity may lie upstream of or parallel to the insulin pathway to impact lifespan and healthspan.

Overall, our results support that DAF-16 positively regulates *pck-2*, which is required to extend *C*. *elegans* healthspan, and suggest that specific cells in the anterior and posterior intestine are central to this regulation. DAF-16 thus plays a critical role in promoting gluconeogenesis and healthy aging at least in part by transcriptional regulation of key biosynthetic enzyme cytoplasmic PCK-2.

### Expression of transcriptional fusion *P*_*pck-2*_*gfp* is induced under food limitation via a *daf-16*-dependent mechanism

**Fig 4:**
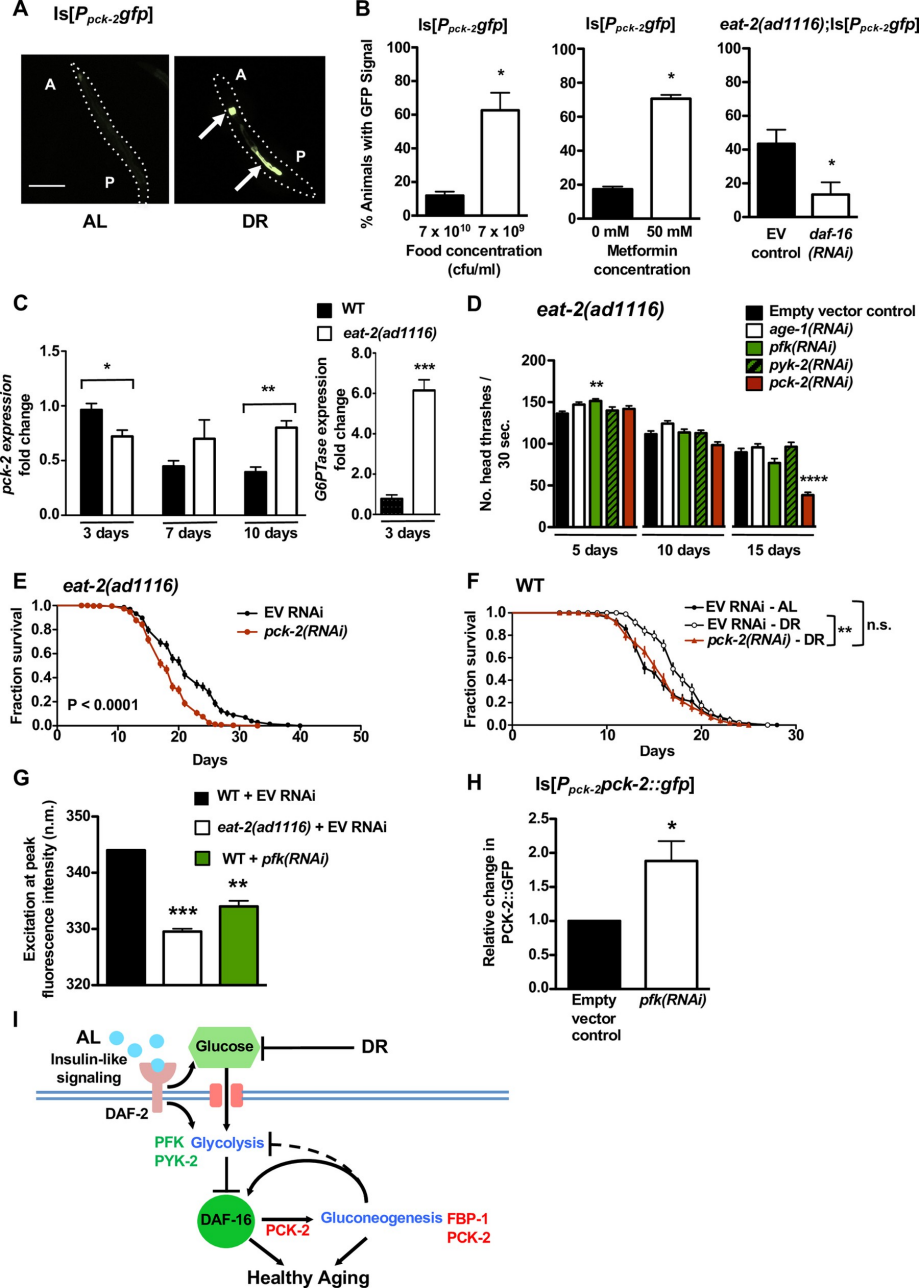
Dietary Restriction Induces Gluconeogenic Gene *pck-2* Expression via DAF-16, and *pck-2* Expression is Needed for DR-associated Healthspan Benefits. (A) Representative wild-type animals expressing a GFP transcriptional reporter for *pck-2* (*pck-2* promoter GFP fusion, lacking *pck-2* coding sequences) under *ad libitum* (AL) or dietary restricted (DR) conditions, day 4 of life. DR animals were raised on diluted (7 x 10^9^ cfu/ml) OP50; AL is undiluted (7 x 10^10^ cfu/ml) bacteria. A = anterior, P = posterior, intestinal GFP fluorescence indicated by arrows. Quantitation of GFP observations is described in (B). Bar, 200 μm. (B) *pck-2* transcriptional reporter GFP expression under three distinct food limitation conditions. Left: as in (A), wild-type animals expressing the *P*_*pck-2*_*gfp* reporter were raised on plates seeded with undiluted (7 x 10^10^ cfu/ml) or diluted (7 x 10^9^ cfu/ml) bacteria and the number of animals displaying GFP fluorescence was scored on day 4 of life. Ampicillin was added to plates to inhibit bacteria growth. The number of animals with GFP fluorescence increases with bacterial dilution (*P* = 0.009, unpaired t test). Data are pooled averages from 3 independent trials, n = 10 animals per condition per trial. Error bar represents SEM. * *P* < 0.05. Center: metformin is a drug used to treat type-2 diabetes that induces features of dietary restriction-like metabolism in *C*. *elegans* [41]. Animals expressing *P*_*pck-2*_*gfp* were raised from the egg stage on plates containing either no metformin or 50 mM metformin, and GFP expression was observed on day 4 of life. The number of animals with GFP expression increases on 50 mM metformin plates (*P* = 0.04, unpaired t test). Data are pooled averages from 3 independent trials, n = 60 animals per condition per trial. Error bar represents SEM. * *P* < 0.05. Right: GFP fluorescence in *eat-2(ad1116)* mutants expressing *P*_*pck-2*_*gfp* and treated with either an empty vector control RNAi or *daf-16(RNAi)*. Under DR, *daf-16(RNAi)* reduces the frequency of observing GFP expression on all tested days (data shown are from day 8 of life, *P* = 0.05, unpaired t test). Wild-type animals carrying Is[*P*_*pck-2*_*gfp*] showed no GFP signal on day 8. Data are pooled averages from 3 independent trials, n = 60 animals per condition per trial. Error bar represents SEM. * *P* < 0.05. (C) Left-hand graph: qRT/PCR-evaluated *pck-2* expression levels from early-to-late life in wild-type (black bars) and dietary-restricted (white bars) *eat-2(ad1116)* mutants. While *pck-2* transcript levels are lower in *eat-2* mutants (vs. wild-type, *P* = 0.007, unpaired t test) early in life, *pck-2* expression remains nearly constant over time in the *eat-2* background (vs. wild-type at 10 days, *P* = 0.0008, unpaired t test). Expression level for all results are represented as fold change with respect to the wild type day 3 level. * *P* < 0.05; ** *P* < 0.005. *P* values were calculated based on three technical repeats per strain per day tested, with approximately 10,000 animals per sample. Right-hand graph: qRT/PCR-evaluated G6P translocase (G6PTase) *F47B8*.*10* expression levels in wild-type and dietary-restricted *eat-2(ad1116)* mutants on day 3 of life. The gluconeogenic-specific enzyme glucose-6-phosphatase is significantly up-regulated in the *eat-2* background (*P* < 0.0005, unpaired t test). *P* values were calculated based on three technical repeats per strain, with approximately 10,000 animals per sample. (D) Swimming rates of *eat-2(ad1116)* mutants disrupted in glycolytic or gluconeogenic activity. Disrupting glycolytic activity with *pfk(RNAi)* results in small but significant increases in swimming rates early in life (day 5; *P* < 0.01, one way ANOVA, a 12% increase) and no later change; glycolytic gene *pyk-2(RNAi)* did not significantly affect swimming ability on any tested day. Disrupting gluconeogenic gene *pck-2* significantly reduces swimming rates on day 15 (*P* < 0.0001 by one way ANOVA, a 58% reduction). Data are pooled from 3 independent trials, n = 40 animals per condition per trial. Error bars represent SEM. ** *P* <0.001; *** *P* < 0.0001. All one way ANOVA analyses performed with Dunnett’s multiple comparisons test. (E) Survival curves of long-lived *eat-2(ad1116)* mutants treated with RNAi against *pck-2*. *pck-2(RNAi)* decreases median survival by 13.79%, and also results in decreased survival (survival difference *P* < 0.0001, Log-rank). Data are pooled from 5 independent trials, n = 60 animals per condition per trial. (F) Survival curves of wild-type animals raised under *ad libitum* (AL) or dietary restriction (DR) conditions, with and without *pck-2(RNAi)*. Median survival of animals expressing the empty vector control was higher under DR conditions as compared to well-fed controls (an 18.62% increase, see S1 Table for details) (survival differences *P* = 0.0039, Log-rank test). Disrupting gluconeogenic gene *pck-2* abolished these beneficial DR effects: animals raised on diluted gluconeogenic RNAi bacteria had survival curves that were not significantly different from those of well-fed animals (n.s., Log-rank). Data are pooled from 2 independent trials, n = 60 animals per condition per trial. ** *P* < 0.005; n.s. = not significant. (G) Excitation wavelengths corresponding to peak age pigment fluorescence intensities in wild-type and *eat-2(ad1116)* dietary restricted mutants raised on empty vector control RNAi, and in wild-type animals treated with RNAi against glycolytic gene *pfk*. Dietary restriction lowers age pigment peak excitation wavelengths, and this shift to lower excitation wavelengths (the ExMax shift) can be used as a biomarker for DR [64]. Like *eat-2* mutants, *pfk(RNAi)* animals show ExMax shifts to lower excitation wavelengths on day 5 of life (the average ExMax value for wild-type animals with *pfk* disruptions is 334.0 nm vs. 344.0 nm for wild-type controls; ExMax values for *eat-2(ad1116)* animals is 330.0 nm; *P* < 0.0001 for *eat-2(ad1116)* + vector control and *P* < 0.001 for WT + *pfk(RNAi)*, one way ANOVA). Data are averages from 2 independent trials, n = 30 animals per condition per trial. Error bars represent SEM. ** *P* < 0.001; *** *P* < 0.001. All one way ANOVA analyses performed with Dunnett’s multiple comparisons test. (H) Wild-type animals expressing Is[*P*_*pck-2*_*pck-2*::*gfp*] and treated with a vector control or with RNAi against glycolytic gene *pfk*. On day 7 of life, *pfk(RNAi)* significantly increases GFP fluorescence in *P*_*pck-2*_*pck-2*::*gfp*-expressing animals, as quantitated in a spectrofluorimeter (*P* = 0.0383, unpaired t test). Data are averages from 3 independent trials, n = 30 animals per condition per trial. Error bar represents SEM. * *P* < 0.05. (I) Summary model for interactions of glycolysis, gluconeogenesis, and healthy aging. Under *ad libitum* (AL) conditions, insulin-like signaling promotes glucose transport and processing via glycolysis. Glycolytic activity, in turn, suppresses the healthspan-promoting transcription factor DAF-16 to limit healthy aging. Under dietary restriction (DR) or RNAi disruption of *pfk* or *pyk-2*, glucose levels and glycolytic activity decrease, while DAF-16 is activated to increase transcription of pro-healthspan targets including gluconeogenic gene *pck-2*. Elevated gluconeogenic activity (dependent on DAF-16-promoted PCK-2 expression) acts in a feed-forward capacity to further enhance the gluoconeogenic pathway and may inhibit glycolysis/insulin signaling to promote/maintain DAF-16 signaling and healthy metabolism (inhibition represented by a dashed line).

For our studies of *pck-2* expression we also constructed a *pck-2* transcriptional reporter (Is[*P*_*pck-2*_*gfp*]) in which the *pck-2* promoter is fused to GFP and all *pck-2* coding and intron sequences are missing. We noted a striking difference in GFP expression between the functional PCK-2::GFP translational fusion and the P_*pck-2*_ transcriptional reporter: while the PCK-2::GFP translational fusion is constitutively expressed, the *pck-2* promoter-only transcriptional reporter is expressed solely in the intestine and only under food limitation conditions (Fig 4A and 4B). We tested multiple food limitation regimens published to induce dietary restriction-like metabolism (absence of food, food dilution, metformin administration, and the *eat-2* feeding-impaired mutant) to document that animals carrying the *P*_*pck-2*_*gfp* transcriptional reporter exhibit strong GFP fluorescence in the anterior and posterior intestine cells (Fig 4A and 4B) (similar to the constitutive Is[*P*_*pck-2*_*pck-2*::*gfp*] intestinal cell expression pattern (Fig 3B); expression in other cells that express *P*_*pck-2*_*pck-2*::*gfp* is not evident for Is[*P*_*pck-2*_*gfp*]). Strikingly, however, under *ad lib* conditions little, if any, *P*_*pck-2*_*gfp* expression is apparent in anterior or posterior intestinal cells. These data raise the possibility of enhanced *pck-2* expression under food limitation conditions. Increased intestinal GFP produced from the *P*_*pck-2*_*gfp* transcriptional reporter in the DR-mimetic *eat-2* mutant requires *daf-16* (Fig 4B). Thus, food limitation is associated with *daf-16*-dependent transcriptional expression of Is[*P*_*pck-2*_*gfp*] in specific intestinal cells.

We confirmed an increase in native *pck-2* transcripts in the *eat-2* DR-mimetic mutant relative to WT later in life by qPCR analysis (Fig 4C). While *pck-2* expression decreases in wild-type animals as they age (0.44 and 0.39 fold change as compared to the WT day 3 level for days 7 and 10, respectively), *pck-2* levels remain remarkably level throughout the *eat-2* lifespan (0.72, 0.70 and 0.80 fold change as compared to WT day 3 level for day 3, 7, and 10, respectively) (Fig 4C). Notably, *pck-2* transcript levels under DR are elevated in mid-to-late life when compared to *ad lib* fed animals, just as we see with the *P*_*pck-2*_*gfp* reporter (Fig 4B). Levels of the gluconeogenic gene encoding the *C*. *elegans* glucose-6-phosphate translocase ortholog are also increased under *eat-2* DR (Fig 4C), suggesting that DR metabolism may generally feature elevated transcripts of the enzymes of gluconeogenesis, with consequent increased gluconeogenic capacity.

The difference in expression profiles between full length translational fusion (constitutive gut expression) and the transcriptional fusion (food limitation-induced gut expression) might be attributed to different sequence inclusions in the transcription and translational constructs, although an alternative possibility can be considered, grounded in the fact that the translational fusion constitutes a PEPCK over-expression situation. Because in WT, qPCR shows that *pck-2* transcript levels decline with age, but in the *eat-2* DR model *pck-2* transcript levels remain elevated (Fig 4C), it is tempting to speculate that the elevation of PCK-2 protein associated with the over-expressed functional translational fusion might be the consequence of increased PCK-2 activity that acts in a positive feedback loop to maintain a gluconeogenic metabolic state (see Discussion).

### Gluconeogenic PCK-2 is required for the health benefits of DR

Our observations raised the question as to whether PCK-2, induced under DR, is required for the healthspan benefits associated with the DR state. To address this question, we measured locomotion and median survival in DR-constitutive *eat-2* mutants treated with RNAi against *pck-2*. We found significant decreases in swimming rates in *eat-2* mutants for *pck-2* disruptions in late-life (Fig 4D). Moreover, we found that the increased median survival of both *eat-2* DR constitutive mutants and of wild-type animals grown under DR is fully dependent on *pck-2* (Fig 4E and 4F and S1 Table). These data show that PCK-2 activity is critical for benefits induced by two distinct DR-inducing conditions. Together, our data support a model in which DAF-16 positively regulates *pck-2* expression under DR or glucose limitation conditions, in a metabolic shift critical for maintaining healthspan. We find that RNAi-mediated knockdown of *fbp-1*, the other unidirectional enzyme that promotes gluconeogenesis, also eliminates longevity phenotypes of *eat-2* and of WT reared under food limitation (Supplemental S5A and S5B Fig). Together, our data support that gluconeogenic flux may be a critical element in maintaining healthy aging in older animals (Fig 1C and 1D) and in healthy DR metabolism.

### Glycolytic disruptions extend healthspan by promoting gluconeogenic activity and DR-like metabolism

Limiting glycolysis promotes DAF-16 activity (Fig 2A), and DAF-16 is needed for healthspan benefits of glycolysis inhibition (Fig 2B and 2C), gluconeogenic gene *pck-*2 overexpression (Fig 3C and 3D), and some forms of dietary restriction [34]. Our findings raise an intriguing question on metabolic flux: do glycolytic disruptions trigger gluconeogenic / DR metabolism to promote healthy aging?

To address this possibility, we first asked whether the locomotory healthspan effects of glycolytic gene disruption might act in the same pathway as locomotory healthspan effects of feeding limitation *eat-2* DR by addressing whether or not an additional (possibly additive) improvement occurs when both perturbations are operative. We find that glycolytic disruption in the *eat-2* background does not increase older age swimming above that recorded for *eat-2* (Fig 4D), consistent with a model in which glycolytic gene disruptions and DR increase locomotory healthspan via the same pathway (although we cannot be certain that ceiling effects might exist in this particular situation).

We next asked whether glycolytic disruption induces additional features of dietary restriction. We previously showed that under multiple DR conditions, the excitation wavelength corresponding to the peak fluorescence of age pigments/lipofuscin shifts to a wavelength lower than any other longevity pathway mutants or any animal grown in abundant food [64]. Indeed, we found that for glycolytic gene disruption *pfk(RNAi)*, the peak age pigment fluorescence excitation wavelength shifts downward, similar to that found in dietary-restricted *eat-2* mutants (Fig 4G) and in other food limitation conditions, suggesting that *pfk(RNAi)* induces a switch to DR-like metabolism. Interestingly, DR prevents the upregulation of glycolytic PFK with age in mammals [30] and inhibits *pfk* expression in *C*. *elegans* [65], supporting that decreased glycolysis-promoting phosphofructokinase activity may be a key and conserved feature of DR metabolism.

Finally, we found that glycolysis disruptions via *pfk(RNAi)* significantly increase gluconeogenic gene *pck-2* expression, as measured by increased PCK-2::GFP (expressed from Is[*P*_*pck-2*_*pck-2*::*gfp*]) levels (Fig 4H). Thus, genetically limiting glycolysis flux increases gluoconeogenic enzyme expression, likely via *daf-16*-mediated loops that direct a yin/yang metabolic balance between glucose utilization and biosynthesis. Our data support a model in which enhanced gluconeogenic metabolism is key to health benefits under dietary restriction and is needed for normal healthy aging, a new concept in healthy metabolism.

## Discussion

We examined the relationship between *C*. *elegans* glucose metabolism pathways and healthy aging, with an emphasis on the maintenance of physical function and overall mid-life survival health outcomes. A high glucose diet accelerates age-associated decline in mobility and survival robustness. Glycolysis exerts a particularly strong negative influence on healthy aging: limiting glycolysis by disrupting one-way glucose degradation steps can result in large old-age locomotory improvements and increases in median lifespan. A key point in our study is focused on the opposing gluconeogenic pathway: gluconeogenic activity is essential for normal healthy aging, as disrupting dedicated gluconeogenic genes induces progeric decline of locomotory activity and compromises survival. Gluconeogenic pathway integrity is also needed for the beneficial effects of dietary restriction. Importantly, interventions that disrupt glycolysis or increase gluconeogenic activity promote healthy physiology in mid-life without markedly extending maximal lifespan, suggesting that these metabolic interventions improve the quality of aging without extending the period of late-life frailty. Data also indicate that expression of the gluconeogenic PEPCK PCK-2 in just a few intestinal cells can modulate animal-wide metabolic state. Overall, our data highlight a novel paradigm in which glycolysis and gluconeogenesis work opposite to one another to influence the maintenance of adult health. We speculate that this metabolic yin/yang underlies major differences in the quality of aging, which if conserved, validates warnings against high sugar diets.

### High glucose accelerates muscle decline across species

Type II diabetes mellitus is associated with accelerated muscle loss during human aging [10–12] and loss of mouse glycolytic muscle is associated with metabolic dysfunction, a consequence that can further compromise glucose homeostasis [66]. We emphasize that a high glucose diet also accelerates *C*. *elegans* locomotory decline, likely reflective of muscle dysfunction. Previous reports on mobility parameters are consistent with a negative impact of glucose on adult mobility maintenance [14, 20, 67]. Moreover, high glucose, which can compromise mammalian heart health [68], also promotes functional decline of *C*. *elegans* cardiac-like pharyngeal muscle. Although the swimming assay we focus on measures functionality rather than muscle integrity *per se*, our data support *C*. *elegans* is a plausible model for development of protective strategies that maintain muscle functionality against the stress of high glucose/high glycolysis metabolism associated with high sugar diets or diabetes [69].

### Glucose processing negatively impacts healthy aging

Lee et al. show that excessive glucose promotes fatty acid synthesis to shorten lifespan, and suggest that glycolytic and fatty acid synthesis pathway activity may result in the accumulation of toxic intermediate metabolites [52]. Consistent with this suggestion, treatment with glycolytic intermediate DHAP reduces lifespan in glucose-fed nematodes, and inhibition of glycolytic enzyme pyruvate kinase increases lifespan both in high-glucose and control conditions [52]. We show that inhibiting glycolysis increases median lifespan and promotes locomotory ability in older animals. A paradox is that glycolytic interventions also would be anticipated to elevate glucose levels, which can be toxic. That inhibiting glycolysis can be beneficial raises the possibility that, in early- and mid-life, the buildup of toxic metabolites from glucose processing (i.e. DHAP) rather than the overall glucose levels has a significant impact on aging quality. Since maximal lifespan does not appear to be changed by glycolytic inhibition, it is possible that glucose build-up could reach toxic levels in older animals. In future studies it will be of interest to measure glucose levels over adult life and to address whether promoting gluconeogenic activity, like inhibiting glycolysis, can mitigate the harmful effects of excess glucose exposure.

### DAF-16/FOXO promotes gluconeogenic metabolism via a mechanism critical for healthy aging

How exactly does glucose metabolism impact aging quality? When glycolysis is active, *daf-16/*FOXO expression is suppressed, with deleterious consequences for locomotory and whole-animal aging [16]. We document that when glycolysis is inhibited, DAF-16 activity increases, enabling direct positive regulation of key gluconeogenic gene *pck-2*. Without *pck-2* increase, aging is accelerated. Thus, our data suggest that one mechanism by which DAF-16 promotes healthspan is by engaging gluconeogenic pathways via increasing PCK-2 production. This model is supported by chromatin profiling studies showing that *pck-2* is a direct target of DAF-16 [70] and our demonstration that DAF-16 consensus binding sequences are required for *in vivo* effects of *pck-2* expression. Since mammalian DAF-16 ortholog FOXO1 targets PEPCK to regulate hepatic gluconeogenesis [71, 72], our data support that DAF-16/FOXO regulation of *pck-2* may be a facet of a conserved mechanism that controls glucose levels.

### Different roles of PEPCK isoforms as drivers of healthy maintenance: PCK-2 in intestine as a key metabolic regulatory point for healthy aging

The overall impact of gluconeogenic pathway flux in *C*. *elegans* is likely carried out by two distinct PEPCKs, with critical points of action in different tissues. There are three PEPCK genes encoded by the *C*. *elegans* genome. *pck-1* encodes a cytoplasmic PEPCK-C that contributes 85% of the measurable PEPCK activity in young *C*. *elegans* (*pck-2* appears to contribute the rest of measurable PEPCK activity at this time) [59]. PCK-1 is expressed in bodywall muscle, pharyngeal muscle and intestine, and has been suggested to be the workhorse energy/metabolic modulator PEPCK in *C*. *elegans* [59, 73]. Overexpression of *pck-1* from its native promoter [59] or only in bodywall muscle is sufficient to increase overall lifespan (expression of *pck-1* in intestine is not effective; [73]). Disruption of *pck-1* can shorten lifespan [73], although we (S1 Table) do not find this outcome for RNAi interventions (technical differences likely explain the outcomes).

Interestingly, *pck-2*, which we show is another PEPCK-C homolog, seems to serve a different role from *pck-1* in promoting adult health. The restriction of *pck-2* expression to the very anterior and posterior gut in adults suggests a non-autonomous instructive role for PCK-2 in locomotory aging, and aging of the whole animal. More specifically, although a *pck-2* translational reporter (native promoter + whole protein GFP tagged) is expressed in multiple tissue types including the muscles and intestine, treatment with *daf-16(RNAi)* or altering the DAF-16 binding site in the *pck-2* promoter abolishes PCK-2 expression only in particular cells of the intestine, yet this deficit is sufficient to disrupt health benefits. Although GFP-based transgene reporters are not always accurate in reflecting native expression, our finding that adult PCK-2::GFP expression in intestine can drive whole-animal metabolic coordination and hence benefits in locomotion (nerve and muscle) and mid-life survival (system-wide health), implies that metabolic changes in certain gut cells can set the animal-wide maintenance tone. Future studies that express *pck-2* specifically in the intestine in a *pck-2* null background should further test this model.

The specific expression for PCK-2 suggests that metabolic regulation/change in particular sets of cells might constitute a driver mechanism that directs whole-animal changes. PCK-2 might serve as a *daf-16*-dependent first responder that signals for metabolic shift in other cells and tissues. General metabolic director cells would be of interest to identify as their specific manipulation might be targeted for enhanced health maintenance during aging.

### The intestine as a driver for whole animal health

*C*. *elegans* intestine is thought to/appears to perform multiple endodermal activities for the worm, functioning as digestive organ, liver and pancreas. Increasing *daf-16* in intestine upregulates DAF-16 activity in other tissues [74], with FOXO-to-FOXO signaling downregulating intestinal insulin *ins-7* [75]. In flies, overexpressing *dFOXO* specifically in the fat body reduces insulin-like peptide gene *dilp-2* expression in neurons and reduces insulin/IGF-1 signaling in peripheral tissues [76, 77]. Brief FOXO induction in young adult fly intestine is sufficient to extend lifespan [15, 78]. Details of how PEPCK relates to DAF-16 signaling in aging remain to be worked out in worms, flies and possibly mammals, but the possibility of conservation of mechanism has been raised [79].

Indeed, the gut has previously been shown to differentially express food-dependent master regulators of DR [42, 80]. As *pck-*2 is essential for healthspan promotion under DR, the anterior and posterior intestinal cells may monitor food/energy levels and signal non-autonomously to adjust metabolism across the entire body when food becomes limiting. The nature of this signal is not yet known, although it is appealing to speculate that it might parallel mammalian glucagon signaling.

Regarding the question of how PEPCK acts in healthy aging, it might be worth noting that L1 starvation has been demonstrated to use a *daf-16* dependent mechanism to restructure carbohydrate metabolism to drive carbon flux through glyoxylate and gluconeogenesis towards trehalose synthesis [81]. In *C*. *elegans*, excess glucose can be stored as glycogen or in the form of the two-carbon sugar trehalose [20, 82]. Although we have not focused on the energy storage outcome of promoting/preventing gluconeogenesis, high glucose has been shown to increase stored glycogen [20, 82] and genetic disruption of glycogen synthesis induces a *daf-16*-dependent shift toward trehalose elevation associated with longevity and enhanced healthspan [20]. Promotion of gluconeogenesis might be anticipated to enhance trehalose production as has been documented for the *daf-16*-dependent L1 starvation response [81]. In the future, it will be of interest to define how energy stores are impacted by *pck-2*.

### Dietary restriction-mediated longevity requires *pck-2*

Our data show that at least two modes of dietary restriction, genetically induced *eat-2* and food limitation, require *pck-2* expression for healthspan benefits, and that *eat-2* mutants exhibit elevated levels of *pck-2* expression late into life. Our findings interface well with published findings documenting that DR and longevity in general involve a metabolic shift away from glycolysis and toward fatty acid metabolism [59, 61, 83, 84]. A role for the PCK-1 PEPCK-C in DR has been independently documented [73]. We define gluconeogenic metabolism as a critical requirement for DR-associated extensions of healthspan. We also emphasize that, as *pck-2* and *fbp-1* are required to maintain health in older wild-type animals under typical feeding conditions, gluconeogenic activity is not only required for the extended healthspan under DR, but for healthy aging in general.

Together, our results support that promoting gluconeogenic metabolism via PEPCK activity engages a conserved metabolic reorganization that results in significant healthspan benefits. How enhanced gluconeogenesis impacts whole-animal/tissue-specific glucose levels remains to be determined, but a switch to gluconeogenic metabolism may result in a shift to health-promoting storage or utilization of energy stores. For example, gluconeogenesis requires amino acid fuel provided by autophagic proteolysis [85]. Increasing gluconeogenic flux may further promote autophagy, which is required for most longevity pathways (including DR; [86]), and may engage healthy metabolism. That gluconeogenesis can promote healthy aging is supported by studies in other models linking enhanced gluconeogenesis to increased lifespan [87–89], suggesting a conserved pro-longevity mechanism. Interestingly, *pck-2* expression decreases as *C*. *elegans* age [90], but gluconeogenic genes, including *pck-2*, are up-regulated in long-lived dauer and *daf-2* insulin signaling mutants [61, 83, 84]. We also show that *pck-*2 expression remains elevated during adulthood in long-lived *eat-2* DR mutants. We suggest that gluconeogenic capacity, possibly in key cells, is a common component of healthy aging, and compromising this capacity later in life undermines successful maintenance.

### A positive feedback loop for metabolic conditions that promote adult health?

Disrupting glycolytic gene *pfk* can both induce a fluorescent biomarker for the DR state and increase PCK-2 expression, supporting a model in which glycolytic disruptions increase DAF-16 signaling to trigger gluconeogenic/DR metabolism and extend healthspan (Fig 4I). This model is supported by our data showing that DAF-16 is required for the healthspan benefits seen with glycolytic gene disruptions.

Interestingly, although a transcriptional reporter for PCK-2 is expressed in a food-dependent manner, the functional PCK-2 translational reporter is not sensitive to food status. Given published datasets that suggest that elevated PCK-2 transcription levels track with increased longevity/healthspan [61, 83, 84], it seems possible that the high constitutive levels we find when intact *pck-2* is over-expressed reflect a gain-of-activity consequence of high PCK-2. We speculate that high PCK-2 induces a metabolic state in which DAF-16 activity increases, resulting in a positive regulatory loop that stimulates DAF-16 activity and reinforces a healthy metabolic state. As reduced insulin signaling does not require PCK-2 to promote healthy aging via DAF-16, PCK-2 may signal parallel to the insulin pathway to intersect with DAF-16. Alternatively, PCK-2 may increase DAF-16 activity by downregulating insulin signaling, which may lie downstream of PCK-2 in regulating lifespan and healthspan (Fig 4I).

Overall, our data support a model in which nutritional signals engage glucose metabolism to influence the quality of aging via DAF-16 (Fig 4I). Under DR or glucose limitation, increased DAF-16 activity promotes gluconeogenic gene expression, resulting in lifespan and healthspan benefits. When food is plentiful, glycolytic activity effectively suppresses DAF-16, which compromises both locomotory healthspan and lifespan. As high levels of glucose inhibit DAF-16 [16], buildup of toxic metabolites [52] may result from inhibition of gluconeogenic metabolism consequent to elevated glycolytic activity. Our data mechanistically tie high glucose intake with suppression of an anti-aging, pro-healthspan gluconeogenic pathway, underscoring a rationale for limiting dietary glucose. The importance of the gluconeogenesis flux in adult health suggests gluconeogenesis process as a novel target for anti-aging interventions as well as for anti-glucose toxicity approaches.

## Materials and methods

### *C*. *elegans* cultures

The following strains used in this study were provided by the *Caenorhabditis* Genetics Center (CGC, University of Minnesota), which is funded by NIH Office of Research Infrastructure Programs (P40 OD010440): wild-type Bristol N2, TJ1052 *age-1(hx546)*, GR1307 *daf-16(mgDf50)*, DA1116 *eat-2(ad1116)*, RB1966 *R11A5*.*4(ok2586)*, CF1553 N2;muIS84[*P*_*sod-3*_*gfp*]. The following are transgenic lines generated for these studies: ZB4912 bzIs191[*P*_*pck-2*_*gfp P*_*mec-4*_*mCherry*], ZB4913 bzIs192[*P*_*pck-2*_*pck-2*::*gfp P*_*mec-4*_*mCherry*], ZB4914 bzEx283[*P*_*pck-2mut*_*pck-2*::*gfp P*_*mec-4*_*mCherry*]. All strains were grown under standard conditions at 20°C [93] on *Escherichia coli* strain OP50-1 or HT115 for RNAi. All experiments were performed at 20°C. Metformin (Sigma-Aldrich, Catalog # D15,095–9) was added directly to the NGM agar media to a final concentration 50 mM from a 1 M aqueous stock. For high-glucose plates, D-(+)-Glucose (Sigma-Aldrich, Catalog # D9434) was added to the liquid media to a final concentration of 2% or 4% from a 24% aqueous stock.

### Generation of the *fbp-1* (K07A3.1) RNAi clone

As the RNAi feeding vector for *fbp-1* targeting was not available in the Ahringer library [94, 95], we constructed our own vector using the following primers for cloning into the pL4440 plasmid using NheI cut sites: 5’-CGCGCGCTAGCATACGG AATCGCTG-3’ and 5’-GCGCGGCTAGCATCGATTTTTTTTAA-3’.

### Generation of N2;Is[*P*_*pck-2*_*gfp*], N2;Is[*P*_*pck-2*_*pck-2*::*gfp*], and N2;Ex[*P*_*pck-2mut*_*pck-2*::*gfp*] lines

The *P*_*pck-2*_*gfp* and *P*_*pck-2*_*pck-2*::*gfp* vectors were constructed using the In-Fusion HD cloning system (Clontech). For *P*_*pck-2*_*gfp*, we amplified 2.9 kb of sequence upstream of the *pck-2* start codon using the primers 5’-CGACTCTAGAGGATCCGACTGATTGAATGAATGACTGGAG TGTATTGG-3’ and 5’-CCAATCCCGGGGATCCGATTCTCTACACCGACTGTGCCGAAAC TTT-3’ and inserted the product into the pPD95.77 vector (Addgene) linearized with BamHI. For *P*_*pck-2*_*pck-2*::*gfp*, we amplified the *pck-2* coding region and 3’-UTR using the primers 5’-GGAGGACCCTTGAGGGTACCATG TCTGTTGATCCAAACCTTCTTACTCC -3’ and 5’-TCATTTTTTCTACCGGTACCGG CAATGTCTGGACTCTCTTCTCTTGAGC -3’ and inserted the product into the *P*_*pck-2*_*gfp* vector linearized with KpnI. The *P*_*pck-2mut*_*pck-2*::*gfp* vector containing the mutated DAF-16 binding site in the *pck-2* promoter region was generated using the QuikChange II site-directed mutagenesis kit (Agilent) with the *P*_*pck-2*_*pck-2*::*gfp* vector and the following primers: 5'-GAACTGGAGAAAAAAACAAACTTTCCACTTTTCTATAATATGTTCTGCA TTTTCAAAGTTTTTTTCTTAAAGAACATTAACTTTAAT-3' and 5'-ATTAAAGTTAATGTTCTTTA AGAAAAAAACTTTGAAAATGCAGAACATATTATAGAAAAGTGGAAAGTTTGTTTTTTTCTCCAGTTC-3'. All constructs were injected at 50 ng/ul into wild-type animals along with a *P*_*mec-4*_*mCherry* co-injection marker [96] (100 ng/ul). *P*_*pck-2*_*gfp* and *P*_*pck-2*_*pck-2*::*gfp* transgenics were γ-irradiated to identify stably transformed lines as described [97].

### Lifespan assays

Lifespan analyses were performed in the same manner for all strains. For each experiment, except where otherwise noted, the lifespan of 60 animals was measured in each trial. RNAi assays were performed using a feeding library as described [94, 95], with some modifications. For each RNAi clone tested, we placed 15 L4-stage larvae on 3 RNAi plates containing 4 mM IPTG with 5 animals per plate and allowed these to develop to adulthood and then lay eggs over 24 hours. These parental animals were then removed from the plates. 48 hours later, 60 (day 2) L4 larvae were transferred to fresh plates. These animals were transferred to fresh plates every day during the progeny production period, and then every other day thereafter. Animals that did not move when gently prodded were scored as dead. Animals that crawled off the plate or died from vulva bursting or internal hatching were not included in lifespan counts.

### *pck-2* overexpression studies

Studies with the *pck-2* over-expressor (*P*_*pck-2*_*pck-2*::*gfp*) used GFP expressed from the *pck-2* promoter (*P*_*pck-2*_*gfp*) as a control. We used *P*_*pck-2*_*gfp* (lacking the PCK-2 coding sequence) to guard against the possibility that elevating the promoter alone might have biological consequences (such as titrating out transcription factors like DAF-16). Potential effects of having high copy numbers of the *pck-2* promoter would be not be apparent in non-transgenic wild-type animals. In Supplemental S3C Fig, nontransgenic wild-type animals generated during the outcrossing of the transgenic lines were used as controls in lifespan studies with *P*_*pck-2*_*gfp* and *P*_*pck-2*_*pck-2*::*gfp* animals.

### Locomotion assays

Animals were raised from eggs similar to the lifespan assays. 40 individuals were measured for body bend rate in liquid. Briefly, 4 animals were placed in 20 μl M9 buffer on a glass slide and filmed for 30 seconds using a Qimaging Rotera-XR digital camera attached to a dissecting microscope and Streampix imaging software (ver. 3.17.2, NorPix). Swimming rates were calculated using CeleST [92]. Note that although assays in some different panels were sampled at different timepoints in mid-late adulthood due to convenience, locomotory decline progresses over time and comparison was always done to control on the same day. Absolute scores can vary between days/experiments, but relative decline is reproducible. Because baseline scores can vary, our conclusions are only drawn from data from trials within single experiments, not across different experiments.

### Age pigment fluorescence spectroscopy

Age pigment fluorescence intensity was measured as described [64]. Wild-type animals were raised from eggs on plates as in the lifespan assays until day 5 of life. On day 5, 50 animals were transferred to 50 μl 10 mM NaN_3_ solution in a single well of a 96-well white FluoroNunc plate (Nalge Nunc Int’l). The animals were scanned using an *in vivo* spectrofluorimeter (Fluorolog-3, Jobin Yvon Inc., Edison NJ). Peak age pigment fluorescence intensity was determined by scanning through a range of excitation wavelengths from 280–410 nm and an emission wavelength of 430 nm. DataMax data acquisition software (v. 2.20, Jobin Yvon Inc.) and Grams/32 data manipulation software (v. 4.14, Galactic Industries Corp.) were used to process the emission data. Scores are the average age pigment fluorescence intensity levels of three independent trials.

### Pharyngeal pumping assays

Wild-type animals were raised from eggs on NGM control or 2% or 4% glucose plates similar to the lifespan assays. On day 5 of life, the pharyngeal pumping rates of 35 individuals were measured in real time by scoring pharyngeal pumping by eye under a dissecting microscope for 30 seconds.

### DR assays on solid media

For the experiment described in Fig 4A, 4B and 4F and S5 Fig, bleached eggs were placed on plates layered with either undiluted (AL) OP50-1 (for Fig 4A) or HT115 (for Fig 4F) overnight culture or 1:10 (DR) diluted OP50-1 (for Fig 4A) or HT115 (for Fig 4F) culture (the HT115 overnight cultures were grown in the presence of 4 mM IPTG to induce RNAi expression). OP50-1/HT115 were killed immediately after drying on AL and DR plates using 2 x 10^6^ uJ at 254 nm using a Stratagene UV Stratalinker 1800 instrument. For the experiment described in Fig 4B, live OP50-1 cultures were placed on plates containing 100 ug/ml ampicillin to arrest growth at 7 x 10^10^ cfu (AL) and 7 x 10^9^ cfu (DR) concentrations. N2;Is[*P*_*pck-2*_*gfp*] animals were placed on these plates at the L4 stage (day 2 of life) and assays were performed on day 4 of life.

### Quantitation of *P*_*sod-3*_*GFP* and *P*_*pck-2*_*pck-2*::*gfp* expression

N2; muIs84[*P*_*sod-3*_*gfp*] or N2; Is[*P*_*pck-2*_*pck-2*::*gfp*] animals were raised from eggs as in the lifespan assays. On day 5 of life for N2; muIs84[*P*_*sod-3*_*gfp*] and day 7 for N2; Is[*P*_*pck-2*_*pck-2*::*gfp*] animals, we measured GFP fluorescence in 50 animals per condition using a spectrofluorimeter as in the age pigment quantitation assays described above.

### Fluorescence microscopy

N2;Is[*P*_*pck-2*_*gfp*], N2;Is[*P*_*pck-2*_*pck-2*::*gfp*], and N2;Ex[*P*_*pck-2mut*_*pck-2*::*gfp*] animals were raised from eggs on RNAi plates similar to the lifespan assays. On day 7 of life, animals were placed in 10 mM NaN_3_ or M9 liquid media and observed under a 40x objective lens using a Zeiss Axioplan 2 microscope equipped with an X-cite Series 120 (EXPO Photonic Solution, Inc.) fluorescence illuminator. Micrographs were obtained using an Optronics digital microscope camera and Magnafire processing software.

### cDNA synthesis and qPCR

*C*. *elegans* samples (approximately 10,000 animals per sample) were collected by washing using M9 buffer with 0.01% Tween20. To remove residual OP50 *E*. *coli* culture, the worm pellet was applied onto M9 buffer with 10% sucrose solution and spun in a clinical centrifuge at full speed for 1 min. The resulting pellet was transferred into a pre-chilled (with liquid N2) mortar and ground with mortar and pestle. The resulting sample grind was stored at -80°C. Total RNA was extracted from the frozen grind of the worms sample using TRIzol extraction (Life Technology, 15596–026). The resulting sample was treated with DNase I (NEB M0303L) and followed by phenol:chloroform (1,1) purification. The resulting total RNA was re-suspended with dH2O and stored at -80°C. First strand cDNA synthesis was done using SuperScript III RT kit (Life Technology, 18080–044) and using 1ug of the total RNA and oligo(dT)20 for mRNA enrichment. The resulting cDNA samples were used for qPCR. All qPCR reactions were done in triplicate, using KAPA SYBR FAST kit (KAPA Biosystems). Each mRNA level was quantified with reference to the mRNA level of *tba-1* [98]. The primers used for *pck-2* were: CGATATCACCACATGGCTTG and GCTTTCCCAGTCTGGATGAA; for F47B8.10: GCTTCACAAGCTGGGTTCTC and CGAAGACGTACACGGAATGA.

### Mitochondria staining

N2;Is[*P*_*pck-2*_*pck-2*::*gfp*] transgenic animals were raised from eggs on plates similar to the lifespan assays. On day 7 of life, 20 animals were washed and resuspended in 1 μg/ml MitoTracker Red CMXRos (Molecular Probes, M7512) in M9 for 6 hours at 20° C in the dark. Animals were washed four times in M9, then placed on seeded NGM plates for one hour at 20° C in the dark. Mitochondrial staining was imaged as described under Fluorescence Microscopy above.

### Statistical analyses

Log-rank (Mantel-Cox) tests, Gehan-Breslow-Wilcoxon tests, ANOVA, and unpaired t tests were performed using GraphPad Prism version 5.00 for Windows (GraphPad Software, San Diego California).

## Supporting information

S1 Table Lifespan data. These are data described in the main body of the text or for the Supplemental Figures. **“**N total” = the total number of animals monitored in all trials for a particular condition; “N dead” = the number of animals that were counted as dead due to “old age”, i.e. not lost from the assay or due to any developmental or physical defect; “N censored” = number of animals removed from the assay due to causes other than “old age”; these include internal hatching, vulval bursting, or crawling off the plate. AL = ad libitum; DR = dietary restriction. *P* values are from Log-rank tests comparing lifespan curves. The RNAi clones used in this study were sequence confirmed to be specific for their target genes.
(TIF)

S1 Fig Excess glucose availability negatively impacts healthspan of young and old animals; conversely, the benefits of disrupting glycolysis do not include extended maximal lifespan. (A) Swimming rates of wild-type animals raised on control plates or plates containing 4% glucose. Exposure to excess glucose results in significant decreases in swimming rates in young animals (day 5 from hatching; *P* = 0.0004, unpaired t test). n = 40 animals per condition from a single trial; error bars represent SEM; *** *P* < 0.001. (B) Swimming rates of wild-type animals raised on control plates or plates containing 4% glucose. Exposure to excess glucose results in significant decreases in swimming rates late in life (day 13 from hatching; *P* = 0.0062, unpaired t test). n = 40 animals per condition from a single trial; error bars represent SEM; * *P* < 0.01. (C) Swimming rates of wild-type animals raised on control plates or plates containing 2% glucose. Unlike 4% glucose (Figs 1A and S1A and S1B), exposure to 2% glucose does not impact swimming rates on day 8 of life (n.s., not significant, upaired t test). n = 40 animals per condition from a single trial; error bars represent SEM. (D) Maximal lifespan values of WT animals treated with *pfk(RNAi)*, *pyk-2(RNAi)*, and *age-1(RNAi)*. While glycolytic gene disruptions significantly increase median survival (see Fig 1D and S1 Table), maximal lifespans of animals treated with *pfk(RNAi)* or *pyk-2(RNAi)* are not significantly different than those of empty vector (EV) controls (not significant, n.s., by one way ANOVA), suggesting that inhibiting glycolysis specifically promotes mid-life vigor without extending end-of-life frailty. *age-1(RNAi)*, which extends maximal lifespan by inhibiting insulin signaling [99], was used as a positive control. Maximal lifespan data values are averages from 6 independent trials, n = 60 animals per condition per trial (see Fig 1D and S1 Table). **** *P* < 0.0001; one way ANOVA analyses performed with Dunnett’s multiple comparisons test. Maximal lifespan is defined as the mean age at death of the longest-lived 10% of a given population [100].
(TIF)

S2 Fig The healthspan effects of glycolytic and gluconeogenic disruptions are absent in the *age-1* insulin signaling mutant background. (A) Swimming rates of long-lived *age-1* insulin pathway mutants treated with RNAi against glycolytic genes *pfk* and *pyk-2* and gluconeogenic genes *fbp-1* and *pck-2*. Unlike in wild-type (Fig 1C), disrupting glycolysis does not further increase healthspan in the *age-1* background (*pfk(RNAi)* and *pyk-2(RNAi)* vs. empty vector control; n.s., not significant by one way ANOVA), suggesting that inhibiting glycolysis and decreasing insulin signaling might increase healthspan via a common pathway. Also unlike in wild-type, inhibiting gluconeogenesis does not significantly decrease locomotory healthspan in the *age-1* background (*fbp-1(RNAi)* and *pck-2(RNAi)* vs. empty vector control; n.s., not significant by one way ANOVA), demonstrating that reduced insulin signaling can rescue the detrimental healthspan effects seen with gluconeogenic gene disruptions. Data are pooled from 3 independent trials, n = 40 animals per trial. Error bars represent SEM. *age-1(RNAi)* further improves the healthspan of *age-1(hx546)* loss-of-function mutants (*P* = 0.0531 vs. empty vector control; one way ANOVA). n.s. = not significant. All one way ANOVA analyses performed with Dunnett’s multiple comparisons test. (B) Survival curves of *age-1* mutants treated with RNAi against gluconeogenic gene *pck-2*. The harmful effects of *pck-2* RNAi (Fig 1D) are absent in *age-1* mutants: the survival curve is not altered with *pck-2* RNAi treatment (n.s., not significant, Log-rank), and median survival is actually increased by 5.13%.
(TIF)

S3 Fig Expression of a *pck-2* translational reporter increases healthspan measures, and expression of a *pck-2* promoter -only transcriptional reporter does not affect healthspan measures. (A) Wild-type animals expressing the *pck-2* translational reporter on day 7 of life. In the panel on the left, PCK-2::GFP is observed to be in the cytoplasm in posterior cells of the intestine; the panel on the right shows mitochondria in the same intestinal cells stained with the red-fluorescent dye MitoTracker. 24 out of 60 animals displayed this PCK-2::GFP localization pattern on day 7. Bar, 50 μm. (B) Swimming rate profiles of wild-type animals carrying integrated forms of either a transcriptional reporter for *pck-2* (Is[*P*_*pck-2*_*gfp*], “*P*_*pck-2*_*gfp*” lacking *pck-2* coding sequences, used as the control) or *pck-2* expressed from its own promoter from an integrated transgene array (Is[*P*_*pck-2*_*pck-2*::*gfp*], “*pck-2* OE”, used as the over-expressor strain) on day 9 of life. *pck-2* overexpression (OE) significantly increases locomotory ability (*P* < 0.0001, unpaired t test). Data are from a single trial, n = 30 animals per condition. Error bars represent SEM. *** *P* < 0.0005, unpaired t test. (C) Survival curves of wild-type animals expressing a transcriptional reporter for *pck-2* (Is[*P*_*pck-2*_*gfp*], “*P*_*pck-2*_*gfp*”), nontransgenic wild-type siblings generated during the outcrossing of the Is[*P*_*pck-2*_*gfp*] line (“WT Cntl for *P*_*pck-2*_*gfp* Outcross”), *pck-2* expressed from its own promoter (Is[*P*_*pck-2*_*pck-2*::*gfp*], “*pck-2* OE”, used as the over-expressor strain), and wild-type animals generated during the outcrossing of the Is[*P*_*pck-2*_*pck-2*::*gfp*] line (“WT Cntl for *pck-2* OE Outcross”), all raised on *E*. *coli* strain OP50. While the survival curve of the *P*_*pck-2*_*gfp* animals is not significantly different than the curve of nontransgenic wild-type controls (“WT Cntl for *P*_*pck-2*_*gfp* Outcross”; *P* = 0.6283, Log-rank), the curve of the *pck-2* OE animals is significantly right-shifted as compared to both nontransgenic wild-type controls (“WT Cntl for *pck-2* OE Outcross”), and *P*_*pck-2*_*gfp* animals (*P* < 0.0001 for both, Log-rank). Data are pooled from two independent trials, n = 100 animals per line per trial. n.s. = not significant; **** *P* < 0.0001. See S1 Table for details. (D) Swimming rates of wild-type (WT) animals and wild-type animals expressing a *pck-2* transcriptional reporter (WT; Is[*P*_*pck-2*_*gfp*]) on day 11 of life. Swimming rates of the two groups are not significantly different in mid-life (n.s., unpaired t test). Data are from a single trial, n = 30 animals per condition. Error bars represent SEM. n.s. = not significant. (E) Survival curves of wild-type (WT), *pck-2* loss-of-function mutants (*pck-2(ok2586)*), wild-type animals expressing a *pck-2* transcriptional reporter (*P*_*pck-2*_*gfp*), and wild-type animals expressing *pck-2* from its own promoter (*pck-2* OE, used as the over-expressor strain) raised on RNAi *E*. *coli* strain HT115 carrying the empty vector pL4440. *pck-2* mutants have significantly shorter lifespans vs. WT controls (*P* < 0.0001, Log-rank). As in S3C Fig, *P*_*pck-2*_*gfp* animals have a similar survival curve to WT (n.s., not significantly different, Log-rank). Also as in S3C Fig, *pck-2* over-expressors have a survival curve that is significantly right-shifted as compared to *P*_*pck-2*_*gfp* animals (*P* = 0.0092, Log-rank) and to WT (*P* = 0.00118, Log-rank). Data are pooled from 7 independent trials for WT controls; 4 independent trials for *pck-2* mutants; 2 independent trials for *P*_*pck-2*_*gfp* animals; 6 independent trials for *pck-2* over-expressors; approximately 60 animals per trial per strain. See S1 Table for details.
(TIF)

S4 Fig Alignment between *C*. *elegans* glucose-6-Phosphate translocase ortholog and human, mouse, zebrafish, and fly glucose-6-Phosphate exchanger. Alignment of the *C*. *elegans* glucose-6-phosphate translocase ortholog (F47B8.10) with the human glucose-6-phosphate exchanger isoform 1 (E value = 3e-31; 26.56% identity; NCBI reference sequence NP_001157749), mouse glucose-6-phosphate exchanger SLC37A4 isoform b (E value = 5e-30; 25.30% identity; NCBI reference sequence NP_001280559), zebrafish glucose-6-phosphate translocase isoform X1 (E value = 3e-24; 26.48% identity; NCBI reference sequence XP_009289762.1), and Mediterranean fruit fly *Ceratitis capitata* glucose-6-phosphate exchanger SLC37A2 isoform X2 (E value = 3e-04; 23.00% indentity; NCBI reference sequence XP_004522689.1). No ortholog has been documented in *Drosophila melanogaster*. “Cooler” blue colors indicate residues not conserved, while “hotter” red colors indicate increased sequence conservation. Final alignment performed with the PRALINE multiple sequence alignment website (http://www.ibi.vu.nl/programs/pralinewww/). A domain/motif search using Prosite (http://prosite.expasy.org/) reveals a single consensus domain (MFS, or major facilitator superfamily domain, a transmembrane substrate transporter domain), which spans the orthologous sequences.
(TIF)

S5 Fig Gluconeogenic gene *fbp-1* expression is required for healthspan extension under dietary restriction. (A) Survival curves of long-lived, dietary-restricted *eat-2(ad1116)* mutants treated with RNAi against gluconeogenic gene *fbp-1*. *fbp-1*(RNAi) decreases median survival by 10% and shifts the survival curve to the left (*P* < 0.0001, Log-rank). Data are pooled from 5 independent trials, n = 60 animals per condition per trial. (B) Survival curves of wild-type animals raised under *ad libitum* (AL) or dietary restriction (DR) conditions, with and without *fbp-1(RNAi)*. Median survival of animals expressing the vector control was higher under DR conditions as compared to well-fed controls (an 18.62% increase, see S1 Table for details), and survival was increased (*P* = 0.0039, Log-rank test). Disrupting gluconeogenic gene *fbp-1* completely abolished these beneficial DR effects: animals raised on diluted gluconeogenic RNAi bacteria had survival curves that were not significantly (n.s., Log-rank) different from those of well-fed animals. Data are pooled from 2 independent trials, n = 60 animals per condition per trial.w
(TIF)
