# Supplementary figures and images for: Gluconeogenesis and PEPCK are critical components of healthy aging and dietary restriction life extension
Source: PLoS Genet. 2020 Aug 25;16(8):e1008982. doi: 10.1371/journal.pgen.1008982 (PMC7473531; doi:10.1371/journal.pgen.1008982)

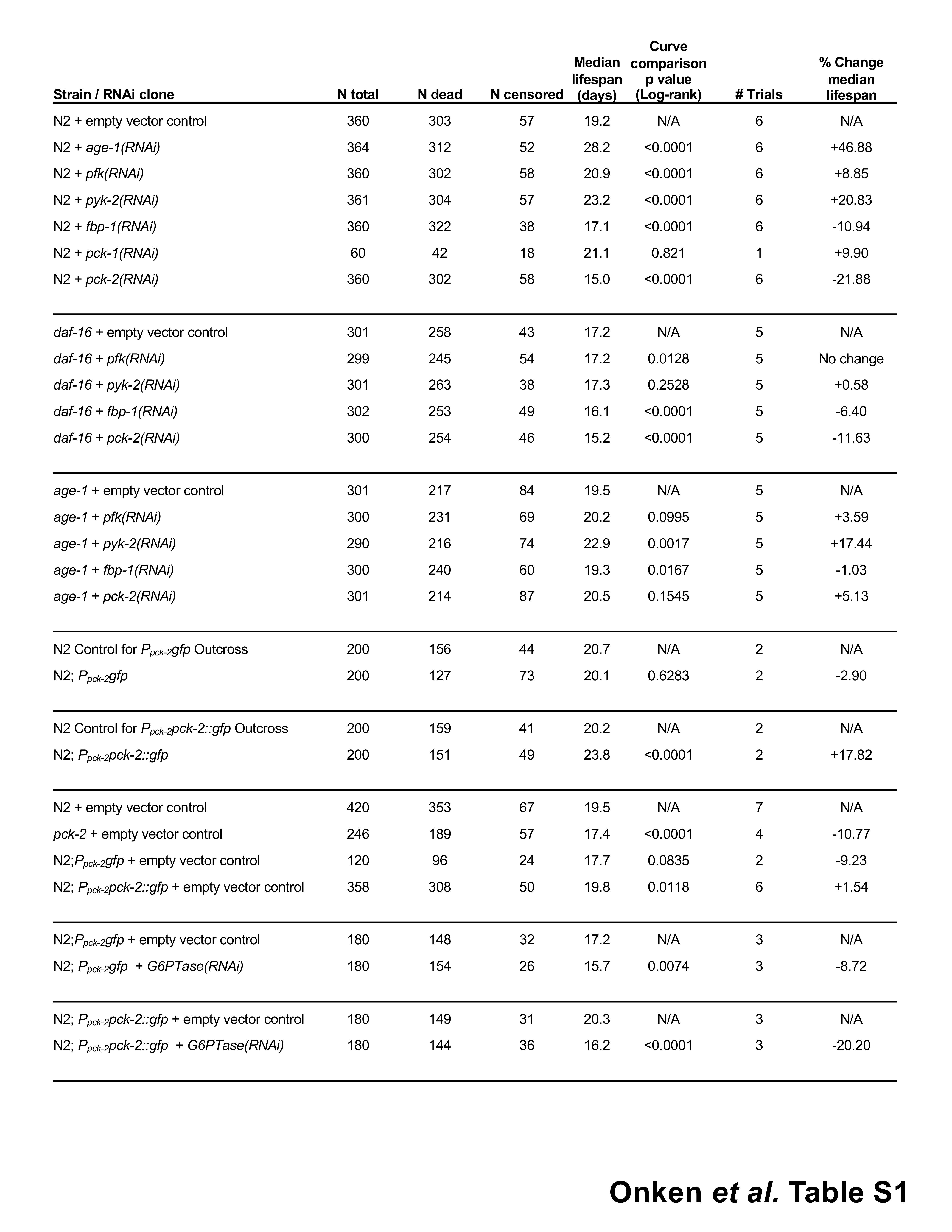

Supplement: S1 Table — These are data described in the main body of the text or for the Supplemental Figures. “N total” = the total number of animals monitored in all trials for a particular condition; “N dead” = the number of animals that were counted as dead due to “old age”, i.e. not lost from the assay or due to any developmental or physical defect; “N censored” = number of animals removed from the assay due to causes other than “old age”; these include internal hatching, vulval bursting, or crawling off the plate. AL = ad libitum; DR = dietary restriction. P values are from Log-rank tests comparing lifespan curves. The RNAi clones used in this study were sequence confirmed to be specific for their target genes. (TIF) [file pgen.1008982.s001.tif]

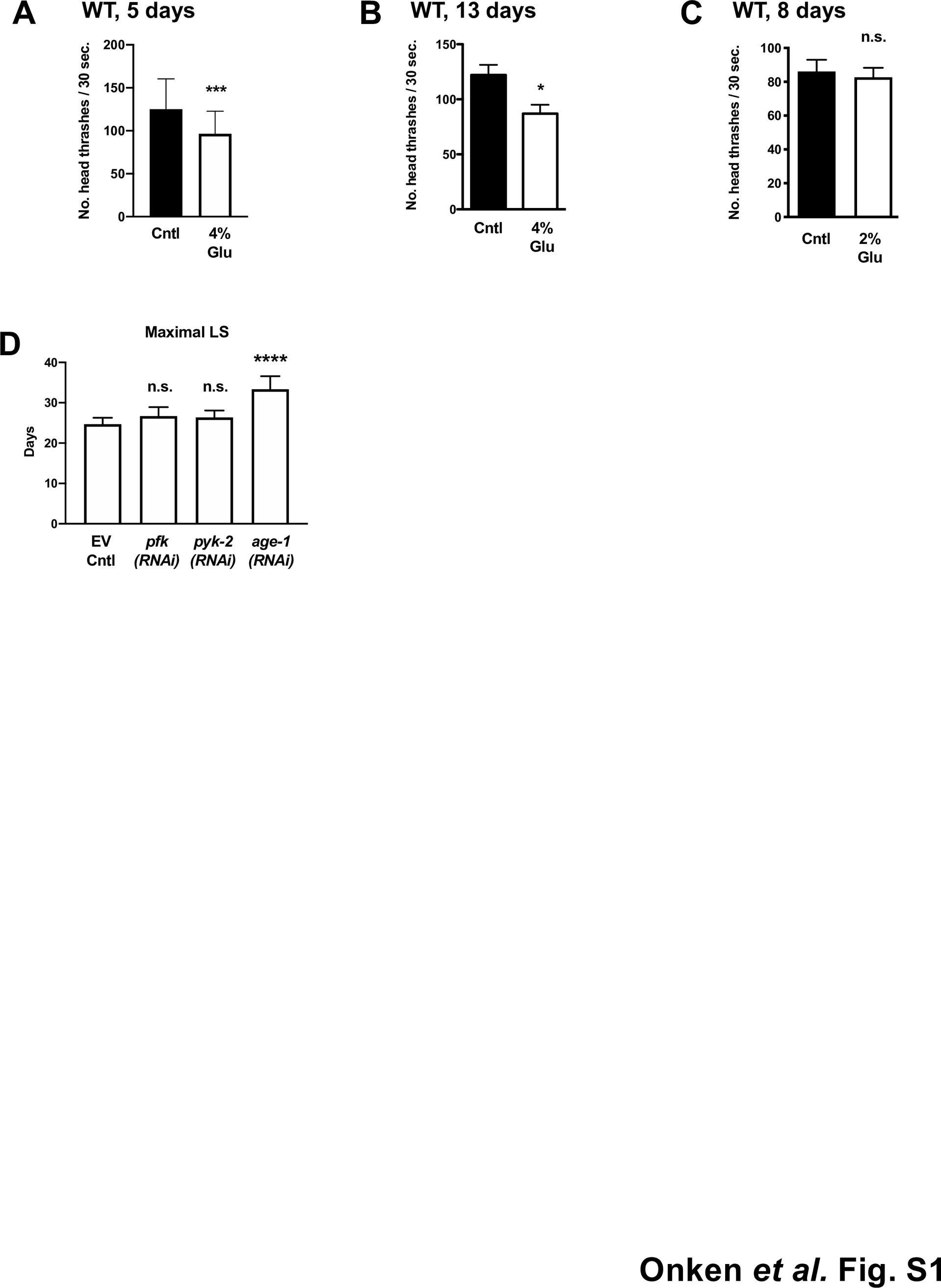

Supplement: S1 Fig — (A) Swimming rates of wild-type animals raised on control plates or plates containing 4% glucose. Exposure to excess glucose results in significant decreases in swimming rates in young animals (day 5 from hatching; P = 0.0004, unpaired t test). n = 40 animals per condition from a single trial; error bars represent SEM; *** P < 0.001. (B) Swimming rates of wild-type animals raised on control plates or plates containing 4% glucose. Exposure to excess glucose results in significant decreases in swimming rates late in life (day 13 from hatching; P = 0.0062, unpaired t test). n = 40 animals per condition from a single trial; error bars represent SEM; * P < 0.01. (C) Swimming rates of wild-type animals raised on control plates or plates containing 2% glucose. Unlike 4% glucose (Figs 1A and S1A and S1B), exposure to 2% glucose does not impact swimming rates on day 8 of life (n.s., not significant, upaired t test). n = 40 animals per condition from a single trial; error bars represent SEM. (D) Maximal lifespan values of WT animals treated with pfk(RNAi), pyk-2(RNAi), and age-1(RNAi). While glycolytic gene disruptions significantly increase median survival (see Fig 1D and S1 Table), maximal lifespans of animals treated with pfk(RNAi) or pyk-2(RNAi) are not significantly different than those of empty vector (EV) controls (not significant, n.s., by one way ANOVA), suggesting that inhibiting glycolysis specifically promotes mid-life vigor without extending end-of-life frailty. age-1(RNAi), which extends maximal lifespan by inhibiting insulin signaling [99], was used as a positive control. Maximal lifespan data values are averages from 6 independent trials, n = 60 animals per condition per trial (see Fig 1D and S1 Table). **** P < 0.0001; one way ANOVA analyses performed with Dunnett’s multiple comparisons test. Maximal lifespan is defined as the mean age at death of the longest-lived 10% of a given population [100]. (TIF) [file pgen.1008982.s002.tif]

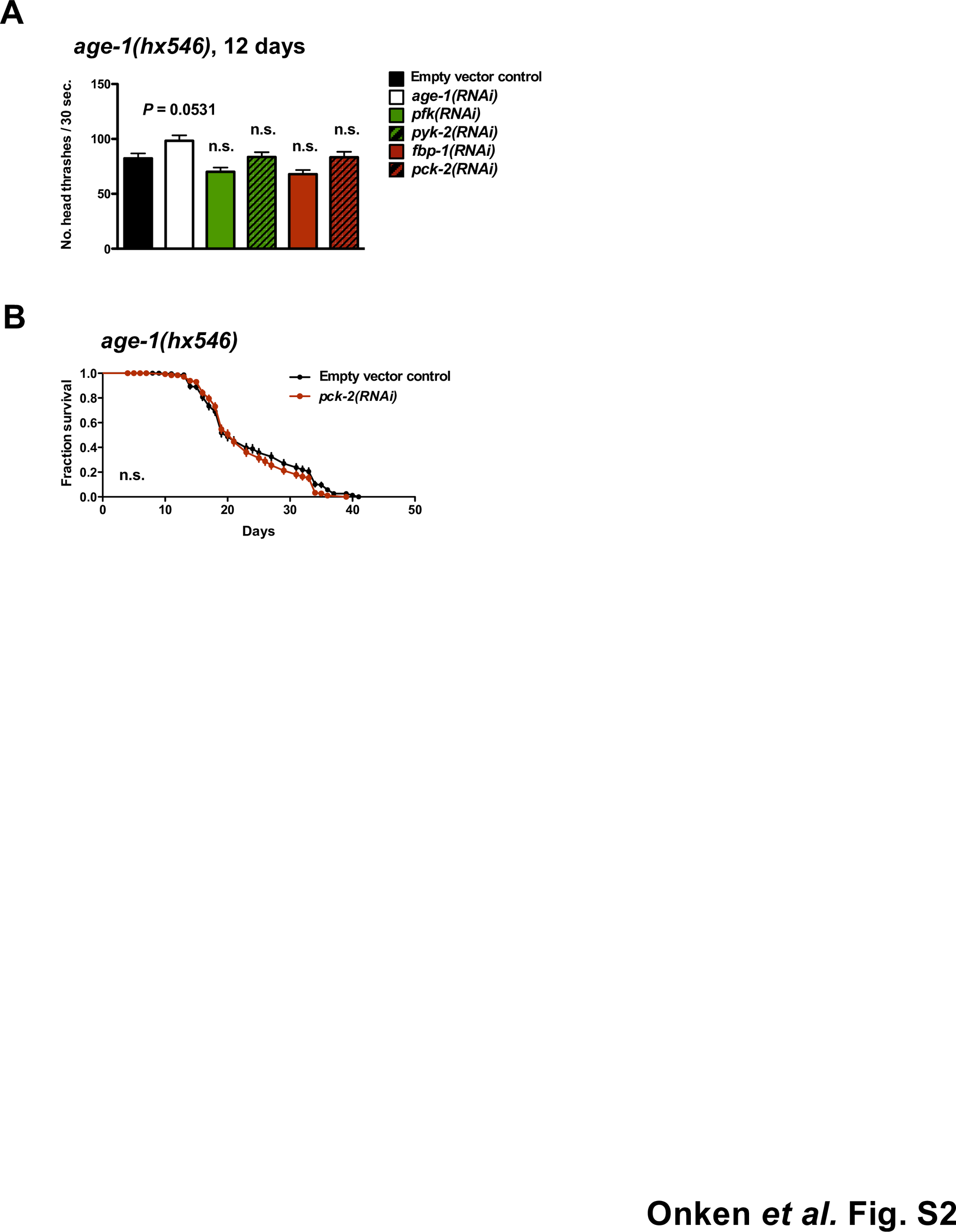

Supplement: S2 Fig — (A) Swimming rates of long-lived age-1 insulin pathway mutants treated with RNAi against glycolytic genes pfk and pyk-2 and gluconeogenic genes fbp-1 and pck-2. Unlike in wild-type (Fig 1C), disrupting glycolysis does not further increase healthspan in the age-1 background (pfk(RNAi) and pyk-2(RNAi) vs. empty vector control; n.s., not significant by one way ANOVA), suggesting that inhibiting glycolysis and decreasing insulin signaling might increase healthspan via a common pathway. Also unlike in wild-type, inhibiting gluconeogenesis does not significantly decrease locomotory healthspan in the age-1 background (fbp-1(RNAi) and pck-2(RNAi) vs. empty vector control; n.s., not significant by one way ANOVA), demonstrating that reduced insulin signaling can rescue the detrimental healthspan effects seen with gluconeogenic gene disruptions. Data are pooled from 3 independent trials, n = 40 animals per trial. Error bars represent SEM. age-1(RNAi) further improves the healthspan of age-1(hx546) loss-of-function mutants (P = 0.0531 vs. empty vector control; one way ANOVA). n.s. = not significant. All one way ANOVA analyses performed with Dunnett’s multiple comparisons test. (B) Survival curves of age-1 mutants treated with RNAi against gluconeogenic gene pck-2. The harmful effects of pck-2 RNAi (Fig 1D) are absent in age-1 mutants: the survival curve is not altered with pck-2 RNAi treatment (n.s., not significant, Log-rank), and median survival is actually increased by 5.13%. (TIF) [file pgen.1008982.s003.tif]

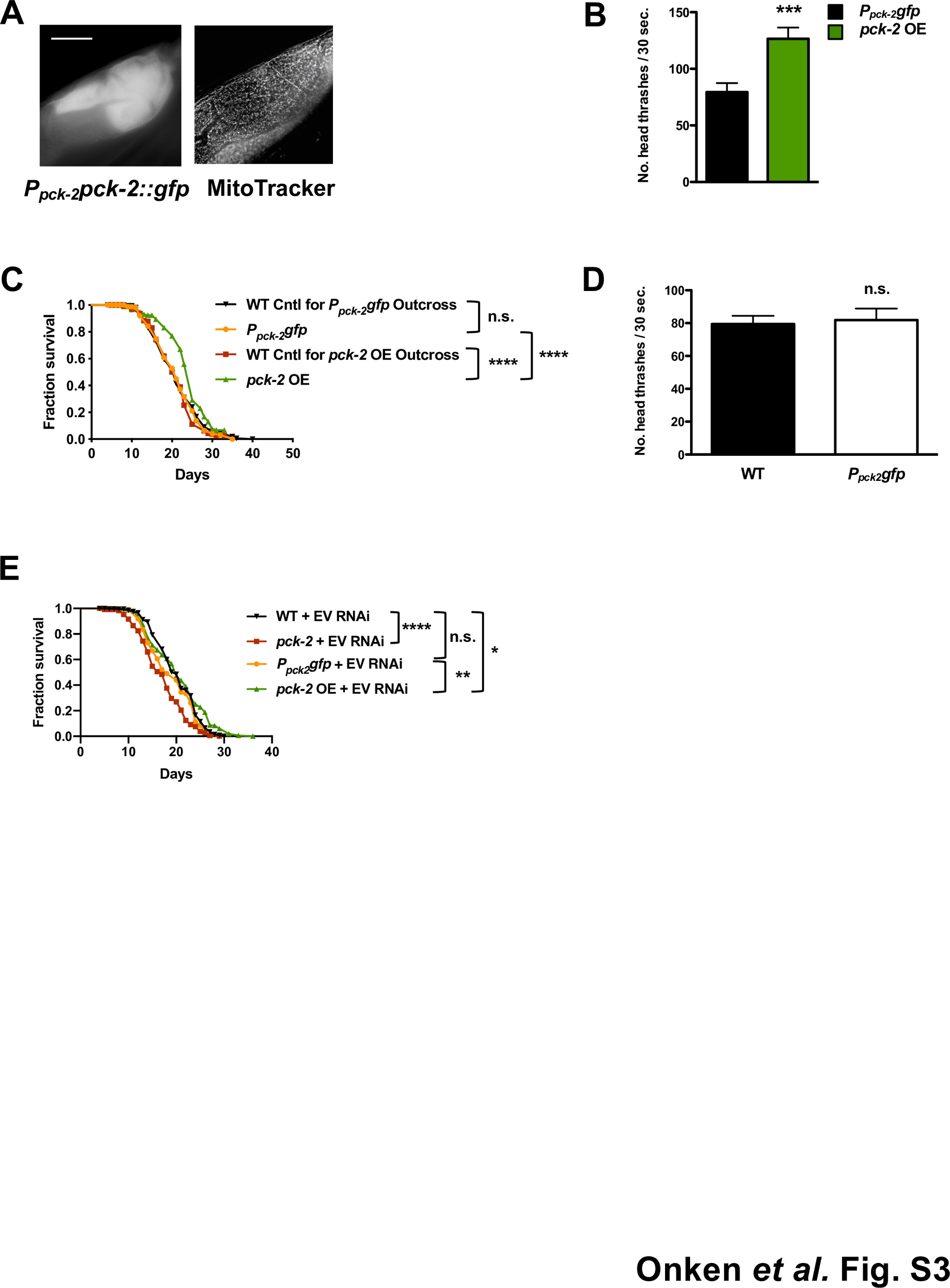

Supplement: S3 Fig — (A) Wild-type animals expressing the pck-2 translational reporter on day 7 of life. In the panel on the left, PCK-2::GFP is observed to be in the cytoplasm in posterior cells of the intestine; the panel on the right shows mitochondria in the same intestinal cells stained with the red-fluorescent dye MitoTracker. 24 out of 60 animals displayed this PCK-2::GFP localization pattern on day 7. Bar, 50 μm. (B) Swimming rate profiles of wild-type animals carrying integrated forms of either a transcriptional reporter for pck-2 (Is[Ppck-2gfp], “Ppck-2gfp” lacking pck-2 coding sequences, used as the control) or pck-2 expressed from its own promoter from an integrated transgene array (Is[Ppck-2pck-2::gfp], “pck-2 OE”, used as the over-expressor strain) on day 9 of life. pck-2 overexpression (OE) significantly increases locomotory ability (P < 0.0001, unpaired t test). Data are from a single trial, n = 30 animals per condition. Error bars represent SEM. *** P < 0.0005, unpaired t test. (C) Survival curves of wild-type animals expressing a transcriptional reporter for pck-2 (Is[Ppck-2gfp], “Ppck-2gfp”), nontransgenic wild-type siblings generated during the outcrossing of the Is[Ppck-2gfp] line (“WT Cntl for Ppck-2gfp Outcross”), pck-2 expressed from its own promoter (Is[Ppck-2pck-2::gfp], “pck-2 OE”, used as the over-expressor strain), and wild-type animals generated during the outcrossing of the Is[Ppck-2pck-2::gfp] line (“WT Cntl for pck-2 OE Outcross”), all raised on E. coli strain OP50. While the survival curve of the Ppck-2gfp animals is not significantly different than the curve of nontransgenic wild-type controls (“WT Cntl for Ppck-2gfp Outcross”; P = 0.6283, Log-rank), the curve of the pck-2 OE animals is significantly right-shifted as compared to both nontransgenic wild-type controls (“WT Cntl for pck-2 OE Outcross”), and Ppck-2gfp animals (P < 0.0001 for both, Log-rank). Data are pooled from two independent trials, n = 100 animals per line per trial. n.s. = not signifi [file pgen.1008982.s004.tif]

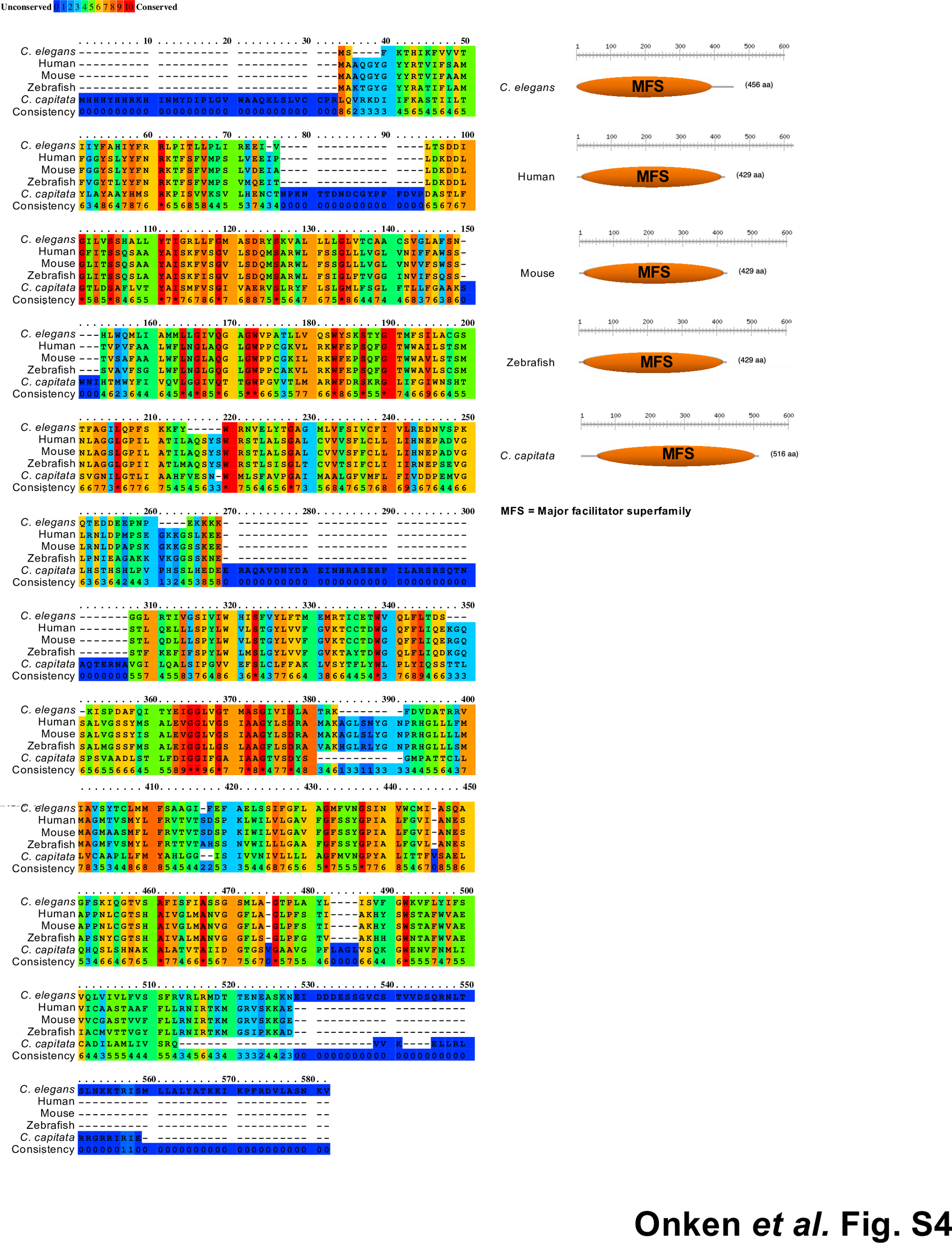

Supplement: S4 Fig — Alignment of the C. elegans glucose-6-phosphate translocase ortholog (F47B8.10) with the human glucose-6-phosphate exchanger isoform 1 (E value = 3e-31; 26.56% identity; NCBI reference sequence NP_001157749), mouse glucose-6-phosphate exchanger SLC37A4 isoform b (E value = 5e-30; 25.30% identity; NCBI reference sequence NP_001280559), zebrafish glucose-6-phosphate translocase isoform X1 (E value = 3e-24; 26.48% identity; NCBI reference sequence XP_009289762.1), and Mediterranean fruit fly Ceratitis capitata glucose-6-phosphate exchanger SLC37A2 isoform X2 (E value = 3e-04; 23.00% indentity; NCBI reference sequence XP_004522689.1). No ortholog has been documented in Drosophila melanogaster. “Cooler” blue colors indicate residues not conserved, while “hotter” red colors indicate increased sequence conservation. Final alignment performed with the PRALINE multiple sequence alignment website (http://www.ibi.vu.nl/programs/pralinewww/). A domain/motif search using Prosite (http://prosite.expasy.org/) reveals a single consensus domain (MFS, or major facilitator superfamily domain, a transmembrane substrate transporter domain), which spans the orthologous sequences. (TIF) [file pgen.1008982.s005.tif]

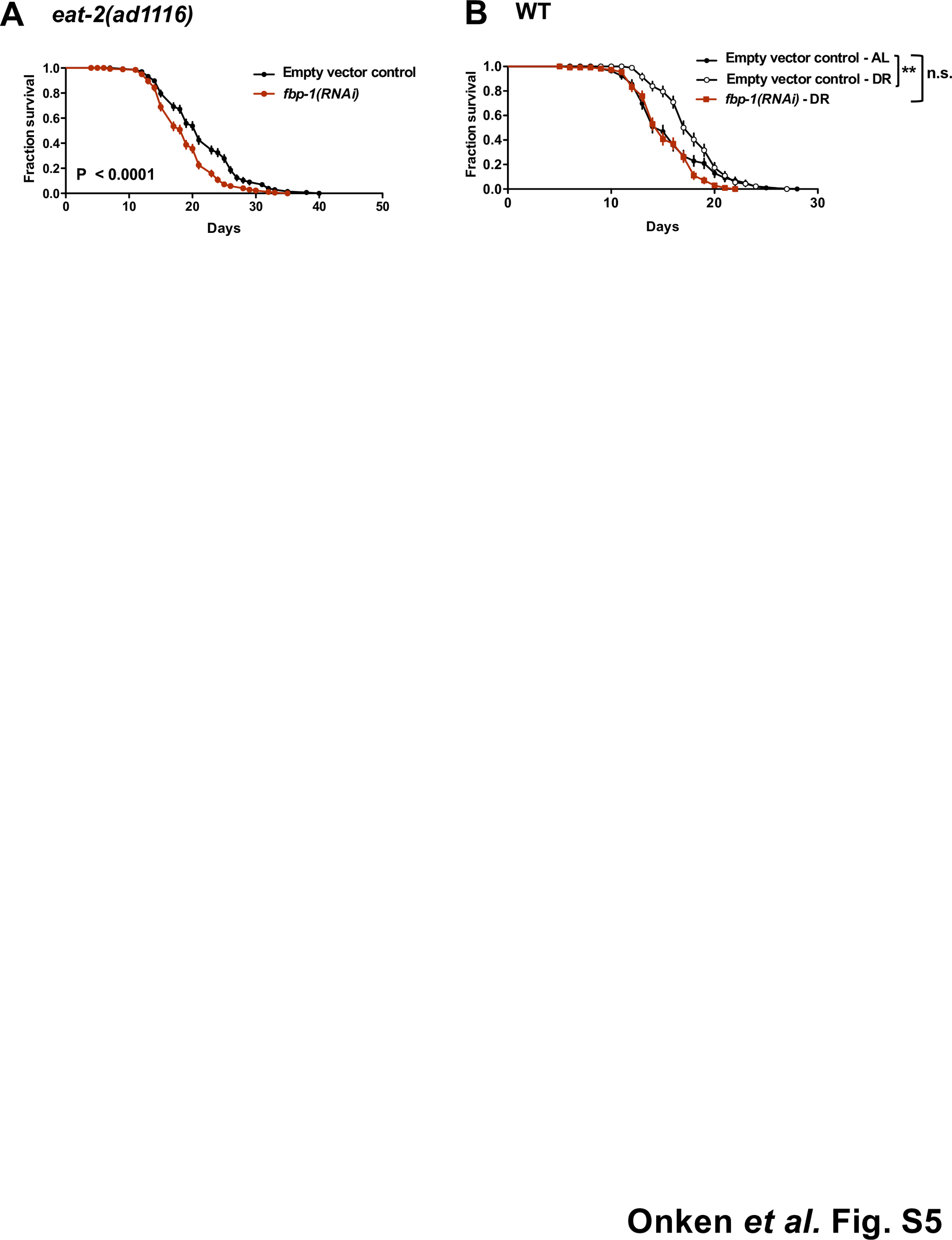

Supplement: S5 Fig — (A) Survival curves of long-lived, dietary-restricted eat-2(ad1116) mutants treated with RNAi against gluconeogenic gene fbp-1. fbp-1(RNAi) decreases median survival by 10% and shifts the survival curve to the left (P < 0.0001, Log-rank). Data are pooled from 5 independent trials, n = 60 animals per condition per trial. (B) Survival curves of wild-type animals raised under ad libitum (AL) or dietary restriction (DR) conditions, with and without fbp-1(RNAi). Median survival of animals expressing the vector control was higher under DR conditions as compared to well-fed controls (an 18.62% increase, see S1 Table for details), and survival was increased (P = 0.0039, Log-rank test). Disrupting gluconeogenic gene fbp-1 completely abolished these beneficial DR effects: animals raised on diluted gluconeogenic RNAi bacteria had survival curves that were not significantly (n.s., Log-rank) different from those of well-fed animals. Data are pooled from 2 independent trials, n = 60 animals per condition per trial.w (TIF) [file pgen.1008982.s006.tif]
